# Supramolecular Modulation of Tumor Microenvironment Through Host−Guest Recognition and Metal Coordination to Potentiate Cancer Chemoimmunotherapy

**DOI:** 10.1002/advs.202408518

**Published:** 2025-01-30

**Authors:** Dan Wu, Jie Zhou, Zhankui Zhang, Yibin Cao, Kunmin Ping, Shaolong Qi, Jianshi Du, Guocan Yu

**Affiliations:** ^1^ College of Materials Science and Engineering Zhejiang University of Technology Hangzhou 310014 P. R. China; ^2^ Vascular Surgery Center The Third Hospital of Jilin University Changchun 130031 P. R. China; ^3^ Ministry of Education Key Laboratory of Bioorganic Phosphorus Chemistry & Chemical Biology Department of Chemistry Tsinghua University Beijing 100084 P. R. China

**Keywords:** host‐guest complex, supramolecular chemistry, non‐covalent interactions, cancer theranostics, tumor microenvironment

## Abstract

The massive amount of indoleamine 2,3‐dioxygenase 1 (IDO‐1) in tumor cells and tumor‐associated immune cells forms a feedback loop that maintains immunosuppressive tumor microenvironment (ITM) and causes immune escape, resulting in the poor prognosis of platinum chemotherapeutics. However, the effective systemic administration of platinum drugs and IDO‐1 inhibitors is strictly limited by their distinct chemical construction, different pharmacokinetic profiles, and heterogeneous distributions. Herein, a novel supramolecular method with the capability to modulate tumor microenvironment is proposed aiming at potentiating the antitumor efficacy of chemoimmunotherapy. Profiting from the dynamic and reversible merits of noncovalent interactions, IDO‐1 inhibitor (IDOi) and 1,2‐diaminocyclohexane‐platinum(II) (DACHPt) are tailor‐encapsulated into supramolecular nanoparticles (SNPs) with the aid of host−guest recognition and metal coordination, respectively, effectively increasing the drug loading and improving their pharmacokinetics. In addition to the authorized chemotherapeutical effect, DACHPt performs a systemic antitumor immune response, which is further magnified by the IDOi‐reversed ITM to encourage T lymphocyte infiltration, guaranteeing long‐term antitumor immune responses to improve cancer prognosis.

## Graphical abstract

A supramolecular modulation of tumor microenvironment is proposed to potentiate cancer chemoimmunotherapy. Based on metal coordination, DACHPt is tailor‐encapsulated into SNPs and evokes an antitumor immune response in the case of a potent chemotherapeutical effect. Meanwhile, high‐efficiency IDOi encapsulation is realized with the help of host−guest complexation, which triggers the maturation of DCs and the renaissance of T lymphocyte recruitment in tumor sites via cutting off the catabolism of Trp and Kyn. This supramolecular synergism of IDOi and DACHPt not only achieves remarkable antitumor efficacy in vitro and in vivo but also guarantees long‐term antitumor immune responses to improve tumor prognosis.

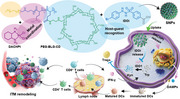

## 1 Introduction

The suppressive metabolic tumor microenvironment, such as hypoxia, acidity, and abnormal tryptophan consumption, greatly restrains the efficacy of cytotoxicity T lymphocytes (CTLs)‐based antitumor response and impairs the tumor immunogenicity, which enables tumor cells to escape immune attack, thus resulting in the failure of platinum chemotherapeutics.^[^ ^1^, ^2^, ^3^, ^4^, ^5^, ^6^ ^]^ Notably, endogenous immunomodulator indoleamine 2,3‐dioxygenase 1 (IDO‐1) upregulated by CTLs‐secreted interferon‐γ (IFN‐γ), restricts the activity of CTLs through initiating a metabolism of tryptophan (Trp) into kynurenine (Kyn).^[^ ^7^, ^8^, ^9^, ^10^ ^]^ Moreover, the accumulation of Kyn accelerates the intratumoral recruitment of regulatory T cells (Tregs), further suppressing the antitumor activity of CTLs.^[^ ^11^, ^12^, ^13^, ^14^, ^15^, ^16^, ^17^, ^18^ ^]^ The IDO‐1 inhibitors‐mediated remodeling of immunosuppressive tumor microenvironment (ITM) is regarded as a promising therapeutic strategy to break the bottlenecks of platinum drugs.^[^ ^19^, ^20^, ^21^ ^]^ However, effective systemic administration of platinum drugs and IDO‐1 inhibitors is strictly limited owing to their distinct chemical construction, different pharmacokinetics profiles as well as heterogeneous distributions. Tailor‐made loading strategies for systemic administration of platinum drugs and IDO‐1 inhibitors are urgently developed to implement ITM remodeling‐enhanced cancer therapy.^[^ ^22^, ^23^, ^24^ ^]^

Supramolecular chemistry, the chemistry beyond molecule, primes sophisticated strategies to manufacture novel delivery systems in possession of the ability to encapsulate therapeutics with unique physicochemical properties.^[^ ^25^, ^26^, ^27^, ^28^ ^]^ The dynamic and reversible features of noncovalent interactions display incomparable superiorities to ensure drug release at the sites of action, dramatically lowering the undesired side effects of therapeutic drugs.^[^ ^29^, ^30^, ^31^, ^32^, ^33^ ^]^ *β*‐cyclodextrins (*β*‐CD), a Food and Drug Administration (FDA)‐approved macrocyclic molecule with a hydrophobic cavity, exhibits the ability to include hydrophobic IDO‐1 inhibitors in its interior cavity via host–guest complexation, thus dramatically improving their bioavailability and stability for biomedical applications. In addition, metal coordinations are suitable to complex platinum drugs through the judicious choice of organic ligands to solve their therapeutic obstacles including low water solubility, poor pharmacological behaviors, and severe side effects.^[^ ^34^, ^35^ ^]^ Therefore, it is of great value to take advantage of supramolecular strategy to realize IDO‐1 pathway blocking‐amplified platinum chemotherapeutics.

**Scheme 1:**
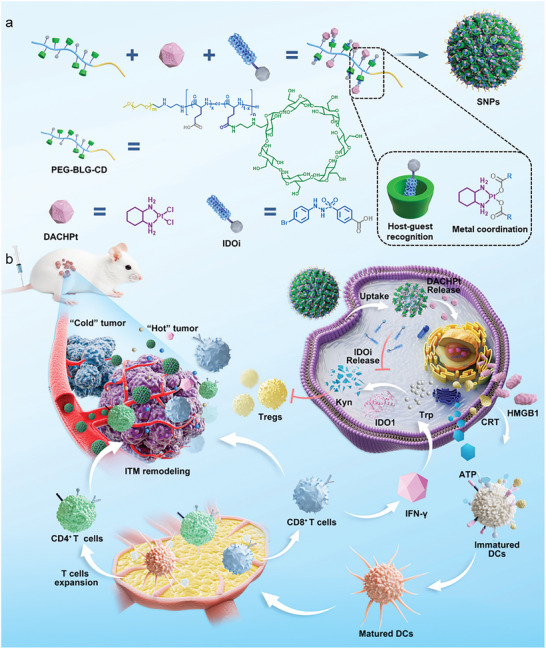
a) Cartoon illustration of the fabrication process of supramolecular nanoparticles (SNPs). b) Systematic illustration of the proposed supramolecular method with the ability to modulate tumor microenvironment to enhance tumor chemoimmunotherapy. DACHPt and IDOi are delivered into tumor cells with the aid of host−guest recognition and metal coordination. Except for the chemotherapeutic effect, DACHPt induces the ICD of tumor cells, which promotes the release of damage‐associated molecular patterns (DAMPs), including high mobility group box 1 (HMGB1), calreticulin (CRT) and adenosine triphosphate (ATP). Immunomodulator IDOi cuts off the IDO‐1 pathway, directly reversing the ITM. The combination of chemotherapy and ITM remodeling not only achieves excellent antitumor efficacy but also revitalizes T lymphocyte recruitment, which transforms “cold” tumors into “hot” tumors and eventually acquires long‐term antitumor immune responses.

Herein, a supramolecular method possessing the ability to remodel tumor microenvironment is proposed to potentiate cancer chemoimmunotherapy (**Scheme** **1**). The polypeptide copolymer carrier (PEG‐BLG‐CD) with stuck carboxy and *β*‐CD units on the side chains, is synthesized via a lithium bis(trimethylsilyl)amide (LiHMDS)‐initiated ring‐opening polymerization (ROP). The unique cavity of additive *β*‐CD shows a strong complexation with IDO‐1 inhibitor (IDOi) and the negatively charged carboxyl groups exhibit a potent ability to coordinate chemotherapeutic 1,2‐diaminocyclohexane‐platinum(II) (DACHPt). With the help of host−guest recognition and metal coordination, the drug‐loading contents of DACHPt and IDOi are significantly improved. In addition to the anticancer capability, DACHPt is capable of inducing the immunogenic cell death (ICD) of tumor cells,^[^ ^36^, ^37^, ^38^, ^39^ ^]^ which activates the antitumor immune responses. Meanwhile, IDOi directly cuts off the IDO‐1 pathway, displaying an extraordinary remodeling ability toward the ITM. The combination of DACHPt and IDOi not only achieves excellent antitumor efficacy in vitro and in vivo, but also revitalizes T lymphocyte recruitment, which is certified by the maturation of dendritic cells (DCs), infiltration of T lymphocytes, suppression of immunosuppressive Tregs and secretion of immunocompetent cytokines, ensuring long‐term antitumor immune responses to preclude tumor recurrence. This tumor microenvironment‐modulated supramolecular strategy pioneers a novel approach to improve cancer prognosis.

## 2 Results and Discussion

**Figure 1:**
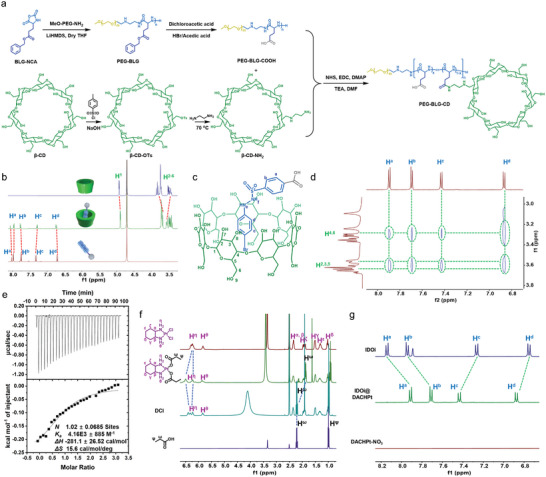
a) Synthetic route of PEG‐BLG‐CD. b) ^1^H NMR spectra (400 MHz, 298K, D_2_O) of *β*‐CD (1.00 mm), *β*‐CD (1.00 mm) + IDOi (1.00 mm), and IDOi (1.00 mm). c) Chemical structure of the host−guest complex between 𝛽‐CD and IDOi. d) 2D NOESY spectrum (600 MHz, 298K, DMSO‐*d_6_*) of *β*‐CD (2.00 mm) and IDOi (2.00 mm). e) ITC result of host−guest complexation between *β*‐CD and IDOi. f) ^1^H NMR spectra of DACHPt (1.00 mm), DACHPt (1.00 mm) + sodium propionate (2.00 mm), DACHPt (1.00 mm) + sodium propionate (2.00 mm) + DCl (5.0 µL) and propionic acid (2.00 mm). g) ^1^H NMR spectra of IDOi (2.00 mm), DACHPt‐NO_3_ (1.00 mm) + IDOi (2.00 mm) (IDOi@DACHPt) and DACHPt‐NO_3_ (2.00 mm).

The diblock polypeptide copolymer (PEG‐BLG) was synthesized via ring‐opening polymerization, in which aminated polyethylene glycol (MeO‐PEG‐NH_2_) acted as the initiator and γ‐benzyl‐L‐glutamate‐*N*‐carboxyanhydride (BLG‐NCA) served as the monomer. After deprotection of benzyl groups under acidic conditions, *β*‐CD‐NH_2_ units were conjugated onto the side chains of PEG‐BLG via amidation reaction, affording the final polypeptide carrier (PEG‐BLG‐CD) containing active sites of carboxy and *β*‐CD groups (**Figure** **1**a).

^1^H NMR spectroscopy was engaged to validate the successful synthesis of PEG‐BLG‐CD. As depicted in Figure S5 (Supporting Information), the average number of *β*‐CD units in PEG‐BLG‐CD was calculated to be 5 by comparing the integration of the protons on *β*‐CD with that on the PEG fragment. Furthermore, the average molecular weight (*M*_n_) of PEG‐BLG‐CD was measured to be 9.48 kDa by using gel permeation chromatography (GPC) (Figure S6, Supporting Information), which was in accordance with the result of ^1^H NMR. The maximum inner diameter of the 𝛽‐CD cavity is ≈7.80 Å, thus the IDOi molecule can easily penetrate into the interior cavity of 𝛽‐CD. The host–guest complexation between *β*‐CD and IDOi was identified by ^1^H NMR spectra. As depicted in Figure 1b, the signals of protons on both IDOi and *β*‐CD underwent obvious chemical shift changes due to the formation of an inclusion complex. Besides, 2D NOESY spectra were employed to reveal the relative position of the host and guest molecules in the host−guest complex. Nuclear Overhauser effect (NOE) correlations were observed between the protons H^a‐d^ on IDOi and protons H^2‐6^ on *β*‐CD (Figure 1c,d), indicating that IDOi penetrated into the cavity of *β*‐CD. Furthermore, according to the isothermal titration calorimetry (ITC) experiment, the binding stoichiometry between IDOi and *β*‐CD was calculated to be 1:1, and the association constant (*K*_a_) of inclusion complex was determined to be (4.16 ± 0.89) × 10^3^ M^−1^ (Figure 1e), which is suitable for the fabrication of supramolecular complex in aqueous solution. By using sodium propionate as a model donor, the metal coordination between carboxy groups and DACHPt was explored by ^1^H NMR spectroscopy. As displayed in Figure 1f, the signals of protons H^η^ on DACHPt split after the addition of sodium propionate, confirming the metal coordination between carboxy and DACHPt occurred. However, the split peaks at 6.21 and 6.50 ppm recovered into broad peaks which were similar to those in DACHPt under acidic conditions, revealing that an acid environment can destroy the metal coordination between carboxy groups and DACHPt and thus accelerate the DACHPt release. In addition, sodium propionate in the coordination system turned into propionic acid in an acidic environment, further verifying the acid responsiveness of metal coordination. Besides, the chemical shift of protons H^a,b^ on IDOi downshifted and the chemical shift of protons H^c,d^ on IDOi upshifted (Figure 1g and Figure S7, Supporting Information) upon adding DACHPt, demonstrating that metal coordination also occurred between the carboxy of IDOi and DACHPt. Collectively, DACHPt could be effectively coordinated by the carboxy groups on both PEG‐BLG‐CD and IDOi.

**Figure 2:**
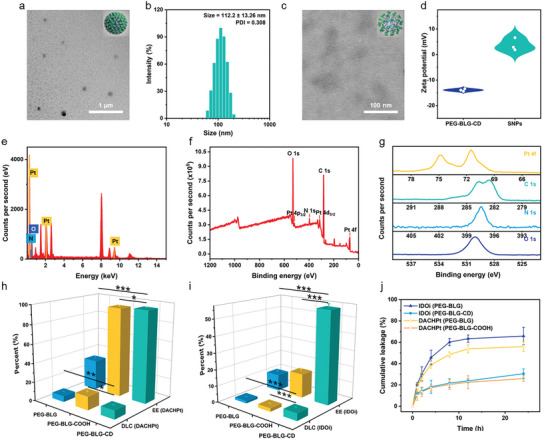
TEM images of SNPs in PBS at pH a) 7.4 and c) 5.5. b) DLS result of SNPs. d) Zeta potential of PEG‐BLG‐CD and SNPs (*n* = 3). e) EDX spectroscopy of SNPs. f,g) XPS spectra of SNPs. The DLC and EE of h) DACHPt and i) IDOi by different carries. j) Drug leakage profiles of the nanoformulations prepared from different carries. Data are presented as mean ± s.d. (*n* = 3). Statistical significance was calculated via ordinary one‐way ANOVA with a Tukey's test. *p < 0.05; **p < 0.01; ***p < 0.001.

Based on the host–guest complexation and metal coordination, SNPs were prepared utilizing a co‐assembly technique. Dynamic light scattering (DLS) and transmission electron microscopy (TEM) were used to reveal the size and morphology of SNPs. As shown in **Figure** **2**a, SNPs showed a homogeneous spherical morphology in an aqueous solution, and its hydrodynamic size was 113 nm (Figure 2b). The Zeta potential of PEG‐BLG‐CD was −13.9 mV, which increased to 3.77 mV upon the formation of SNPs (Figure 2d). Energy‐dispersive X‐ray (EDX) spectroscopy was used to recognize the components of SNPs. As depicted in Figure 2e, the peaks of N, O, and Pt were clearly identified. Besides, the peaks of C (1s), O (1s), N (1s), Pt (4d), Pt (4p) and Pt (4f) were clearly seen in the X‐ray photoelectron spectroscopy (XPS) spectroscopy of SNPs (Figure 2f), and the Pt 4f XPS patterns exhibited peaks at 71.5 eV (Pt 4f_7/2_) and 74.8 eV (Pt 4f_5/2_) (Figure 2g), indicating Pt was in a tetravalent state. The size of SNPs was well maintained for 7 days in PBS (Figure S8, Supporting Information), and little changes in average diameters were found after 24 h of incubation in RPMI 1640 and culture medium (RPMI 1640 containing 10% fetal bovine serum) (Figure S9, Supporting Information), suggesting the preeminent colloidal stability of SNPs in the physiological environment.

Inductively coupled plasma mass spectrometry (ICP‐MS) and high‐performance liquid chromatography (HPLC) were utilized to determine the drug loading capacity of PEG‐BLG‐CD. Compared with PEG‐BLG and PEG‐BLG‐COOH polymers which were not equipped with carboxyl and/or *β*‐CD units, an approximate 3.0‐fold increase in encapsulation efficiency (EE) of DACHPt and IDOi was detected for PEG‐BLG‐CD by fully taking advantage of noncovalent interactions. In addition, the drug loading contents (DLC) of DACHPt and IDOi were also remarkably improved in the group of PEG‐BLG‐CD (Figure 2h,i). These results corroborated that metal coordination and host−guest complexation were the tailor‐made supramolecular strategies to effectively and synergistically encapsulate DACHPt and IDOi with different physicochemical properties. Premature drug leakage during systemic circulation induced by large dilution volume is one practical concern for nanoparticles, which reduces effective drug concentration at the target sites and increases off‐target exposure. As displayed in Figure 2j, a 55.9% DACHPt leakage ratio and a 65.8% IDOi leakage ratio were monitored for the nanoparticles formulated by PEG‐BLG carrier, whereas the DACHPt and IDOi leakage ratios lowered to 25.9% and 30.5%, respectively, after encapsulation into the nanoformulations fabricated by PEG‐BLG‐COOH and PEG‐BLG‐CD carriers, further demonstrating the important roles of noncovalent interactions in enhancing the stability of SNPs.

**Figure 3:**
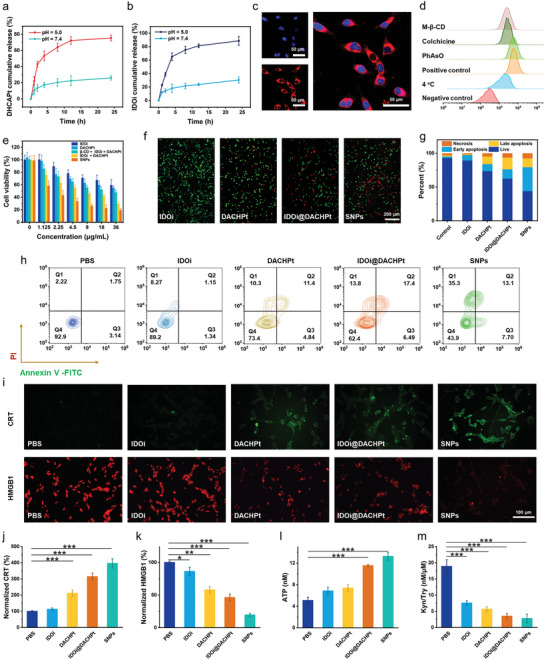
Drug release profiles of a) DACHPt and b) IDOi under different conditions. c) CLSM images of CT26 cells treated with BODIPY‐labeled SNPs for 8 h. d) Cellular internalization of SNPs by CT26 cells in the presence of various inhibitors. e) Cell viability of CT26 cells after different treatments. f) Calcein‐AM/PI staining fluorescence images of CT26 cells after different treatments. g), h) Annexin‐V‐FITC/PI staining flow cytometry analysis of CT26 cells after different treatments. i) CRT exposure and HMGB1 efflux of CT26 cells after different treatments. Micro‐image analysis of the j) CRT exposure and k) HMGB1 efflux of CT26 cells after different treatments. l) ATP level and m) the relative Kyn/Trp ratio in the supernatant of CT26 cells after different treatments. Data are presented as mean ± s.d. (*n* = 3). Statistical significance was calculated via ordinary one‐way ANOVA with a Tukey's test. *p < 0.05; **p < 0.01; ***p < 0.001.

The drug release profiles were monitored at different pH over a period of 24 h. As shown in **Figure** **3**a, <20% of the loaded DACHPt was released at pH 7.4, which proved the high stability of SNPs in the physiological environment. In sharp contrast, 75.4% of DACHPt was released under weak‐acid condition at pH 5.5, disclosing a pH‐responsiveness of SNPs in drug release, the reason of which is attributed to the impaired metal coordination between carboxylate and DACHPt owing to the protonation of carboxylate anion in acid environment. Because the acid‐triggered DACHPt release induces the disassembly of SNPs, the IDOi release may be cascadingly promoted. Exactly, IDOi exhibited a pH‐responsive release profile, in which 90.4% of IDOi was released in an acidic buffer, whereas an inefficient release behavior was detected in a neutral buffer (Figure 3b). Meanwhile, the TEM image showed that SNPs collapsed upon incubation in an acidic environment (Figure 2c; Figure S10, Supporting Information), confirming the acid‐triggered disassembly of SNPs and the release of DACHPt and IDOi. These primary studies validated that SNPs were stable during blood circulation and realized explosive drug release after reaching the acidic tumor microenvironment, which is conducive to decreasing toxic and side effects toward normal tissues.

Confocal laser scanning microscopy (CLSM) was applied to monitor the intracellular internalization of SNPs. As DACHPt and IDOi are non‐fluorescent drugs, a boron dipyrromethene (BODIPY) dye was incorporated into SNPs by taking advantage of metal coordinations (Figure S11, Supporting Information).^[^ ^40^ ^]^ BODIPY‐labeled SNPs successfully illuminated CT26 cells in a time‐dependent manner (Figure S12, Supporting Information) and displayed rapid internalization by cells within 8 h (Figure 3c). Meanwhile, flow cytometry showed a 2.52‐fold enhancement of intracellular fluorescence after 8 h incubation (Figure S13, Supporting Information), a direct evidence to convince the high cellular uptake of SNPs. The endocytic pathway of SNPs was investigated using flow cytometry by pre‐treating the cells with different endocytosis inhibitors. As shown in Figure 3d, a significant inhibition of cellular uptake was observed at 4 °C, implying the internalization of SNPs was energy‐dependent. Furthermore, methyl‐*β*‐CD (M‐*β*‐CD), an inhibitor of caveolae‐mediated endocytosis, and colchicine, an inhibitor of macropinocytosis, induced a 57.0% and 54.1% decrease in the endocytosis, respectively. However, cellular uptake of SNPs was not inhibited by oxophenylarsine (PhAsO), which is an inhibitor of clathrin‐mediated endocytosis. Therefore, SNPs were mainly internalized through the caveolae‐mediated endocytic pathway and macropinocytosis.

The in vitro anticancer efficacy of SNPs was assessed using a 3‐(4′,5′‐dimethylthiazol‐2′‐yl)‐2,5‐diphenyltetrazoliumbromide (MTT) assay. No significant decrease in cell viability was monitored for the cells treated with PEG‐BLG‐CD even when the concentration increased to 1.00 mg mL^−1^ (Figure S14, Supporting Information), illustrating its excellent biocompatibility. SNPs exhibited concentration‐dependent cytotoxicity (Figure 3e), and its half‐maximal inhibitory concentration (IC_50_) was calculated to be 3.49 µg mL^−1^, much lower than those of DACHPt (6.68 µg mL^−1^), IDOi (21.0 µg mL^−1^) and IDOi@DACHPt (4.69 µg mL^−1^). Besides, three other tumor cell lines including 4T1, HeLa, and A549 cells also demonstrated the excellent antitumor activity of SNPs (Figures S15–S17, Supporting Information). In addition, a calcein‐AM/PI costaining assay was conducted to distinguish live and dead cells, respectively. Compared with the PBS group, CT26 cells treated with either IDOi or DACHPt showed moderate cell death, suggesting that these formulations did not induce significant cytotoxicity. Nevertheless, obvious cell death was detected in the group of SNPs (Figure 3f). Annexin V‐FITC/PI dual‐staining assay was further used to explore the manner of cell death. Flow cytometry assay revealed that SNPs mainly induced the late apoptosis and necrosis of tumor cells. Specifically, the percentage of late apoptotic/necrotic cells in the group of SNPs was 20.8%, much higher than the groups treated with IDOi (2.49%) or DACHPt (16.2%) (Figure 3g,h). Meanwhile, fluorescence images indicated that SNPs induced the highest level of late apoptosis and necrosis (Figure S18, Supporting Information).

It has been reported that chemotherapy with oxaliplatin (OXA) elicits immune responses by inducing the ICD of tumor cells. Tumor cells suffering ICD would express CRT on the membrane surface which emits “eat‐me” signals to DCs, and secrete ATP and HMGB1 protein extracellularly which promote DC maturation and antigen presentation to naΪve T cells, all of which synergistically switch the “cold” tumor to the “hot” tumor and consequently enhance the chemotherapeutic efficacy of OXA. We then explored the immunogenicity of DACHPt, which is an OXA analog. Apart from the direct cytotoxic effect, SNPs also promoted the expression of CRT on the cell surface (Figure 3i,j), the transposition of HMGB1 outward the nucleus (Figure 3i,k), and ATP release (Figure 3l), suggesting that SNPs elicited antitumor immunity by inducing ICD‐related DAMPs. IDO‐1 is an IFN‐𝛾 inducible enzyme which can inhibit the activity of CTLs by catalyzing the break‐down of Trp into Kyn. The inhibitory effect of SNPs on the IDO‐1 pathway was assessed on CT26 cells using HPLC and colorimetric assay. The results indicated that SNPs significantly reduced the Kyn to Trp ratio compared with other groups (Figure 3m), suggesting that the inhibitory capacity of IDOi was not diminished after being loaded into SNPs.

**Figure 4:**
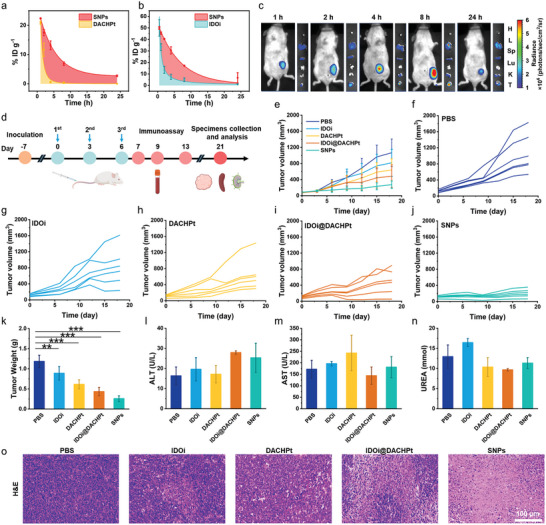
a) Pharmacokinetic behaviors of DACHPt and SNPs determined by ICP‐MS (*n* = 3). b) Pharmacokinetic behaviors of IDOi and SNPs determined by HPLC. c) In vivo imaging of mice and *ex vivo* organ biodistribution at 1, 2, 4, 8, and 24 h post‐injection of BODIPY‐labeled SNPs. d) Therapeutic schedule of SNPs‐mediated tumor therapy. e) Average tumor volume and f‐j) individual tumor growth curves of mice after different treatments (*n* = 6). k) The mean weight of tumors from the mice after different treatments (*n* = 6). l‒n) Routine blood analysis of liver function and kidney function of mice after different treatments (*n* = 3). o) H&E staining of tumor tissues from the mice after different treatments. Data are presented as mean ± s.d. Statistical significance was calculated via ordinary one‐way ANOVA with a Tukey's test. *p < 0.05; **p < 0.01; ***p < 0.001.

Nanoparticles manufactured from block copolymers are extensively employed to amplify the efficacy and alleviate the harmful side effects of drugs, due to their capability to prolong circulation time and facilitate uptake via the enhanced permeability and retention (EPR) effect. The in vivo pharmacokinetics of DACHPt and SNPs were assessed by quantifying the platinum amount in blood using ICP‐MS. The blood circulation half‐life (*t*_1/2_) of SNPs was calculated to be 7.5 h, 4.69 times as that of DACHPt (1.6 h) (**Figure** **4**a). Approximately 2.74% injected dose per gram (ID g^−1^) remained in the plasma at 24 h post‐injection for SNPs, while DACHPt was completely cleared from the plasma at this point. The area under the curve (AUC) of the SNPs was 6.07‐fold greater than that of DACHPt, implying that the blood circulation of SNPs was greatly prolonged through supramolecular nanoformulation. Meanwhile, SNPs displayed a 3.42‐fold longer t_1/2_ and 1.96‐fold greater AUC than those of the free IDOi control (Figure 4b). Furthermore, the in vivo fluorescence imaging was performed at indicated time points post intravenous (i.v.) injection and the major organs and tumor tissue were collected for ex vivo imaging at 24 h post injection. Figure 4c showed that an intensive signal was visible in the tumor area of the mice administered with SNPs at 8 h, and significant intratumoral accumulation was still clearly visible at 24 h post‐injection. Such high tumor specificity was ascribed to the sophisticated supramolecular self‐assembly and the EPR effect of SNPs.

Prior to the assessment of in vivo antitumor efficacy, it is vital to verify that SNPs are safe for i.v. injection. As shown in Figure S19 (Supporting Information), SNPs did not induce apparent hemolysis within the test concentration range (0.125–1.00 mg mL^−1^), implying that SNPs based on host–guest complexation and metal coordination were favorable for in vivo application. Encouraged by the exciting results of in vitro anticancer capability, the in vivo therapeutic efficacy of SNPs was explored using CT26 tumor‐bearing BALB/c mice. The mice were randomly divided into five groups and administrated with (I) PBS, (II) IDOi (10.0 mg kg^−1^), (III) DACHPt (5.0 mg kg^−1^), (IV) IDOi@DACHPt (IDOi, 10.0 mg kg^−1^; DACHPt, 5.0 mg kg^−1^) and (V) SNPs (IDOi, 10.0 mg kg^−1^; DACHPt, 5.0 mg kg^−1^), respectively (Figure 4d). As depicted in Figure 4e–j, Figures S20 and S21 (Supporting Information), the tumor grew madly in the PBS group and the average tumor volume showed a 9.00‐fold increase at day 18. Mice treated with IDOi showed a minimal antitumor capacity, which was comparable to that of the PBS group. Compared with the PBS and IDOi‐treated groups, moderate anti‐tumor efficacy was detected for the mice receiving DACHPt and IDOi@DACHPt treatments. Administration of SNPs strongly suppressed tumor growth. The mean tumor weight of mice in the SNPs group was only 0.26 ± 0.07 g at the end of treatment (Figure 4k), much slighter than those in other groups, demonstrating the excellent antitumor efficacy of the supramolecular nanoplatform. The tumor inhibition ratios of IDOi, DACHPt, IDOi@DACHPt, and SNPs were 25.7%, 45.5%, 60.2%, and 76.6%, respectively. This satisfactory antitumor outcome was supported by the histological analyses, including hematoxylin and eosin (H&E) staining and Ki67 staining. The H&E staining revealed that the tumor tissue from the mice treated with SNPs suffered from the most severe fibrosis, necrosis and karyolysis compared with other groups (Figure 4o). According to Ki67 staining, the lowest level of cell proliferation occurred in the SNPs group (Figure S22, Supporting Information). Neither obvious body weight loss (Figure S23, Supporting Information), major organ damage (Figure S24, Supporting Information) nor nephrotoxicity and hepatotoxicity (Figure 4l–n; Figure S25, Supporting Information) was observed, indicating that SNPs possessed satisfactory biocompatibility and biosafety. On the contrary, the administration of IDOi, DACHPt, and IDOi@DACHPt resulted in a significant weight loss during therapy (Figure S23, Supporting Information), possibly caused by the non‐specific biodistribution of these drugs with small molecular weight.

**Figure 5:**
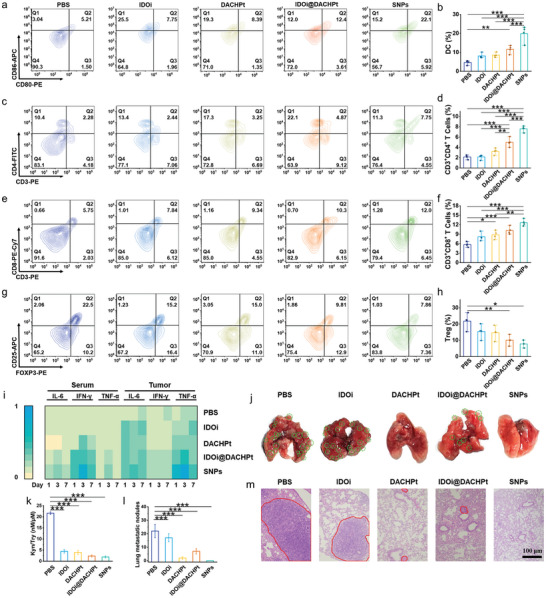
a), b) Flow cytometry analysis of DCs maturation after different treatments. Flow cytometry analysis of c), d) CD3^+^CD4^+^, e), f) CD3^+^CD8^+^ and g), h) CD4^+^CD25^+^FOXP3^+^ T cells in spleens after different treatments. i) The level of immunocompetent cytokines including IL‐6, IFN‐𝛾, and TNF‐𝛼 in serum and tumor tissues after different treatments. j) Photographs of lung tissues from the mice receiving different treatments. k) The relative Kyn/Trp ratio in tumor tissues after different treatments. l) Statistics of the number of lung metastatic nodules. m) H&E staining of lung tissues from the mice after different treatments. Data are presented as mean ± s.d. (*n* = 3). Statistical significance was calculated via ordinary one‐way ANOVA with a Tukey's test. *p < 0.05; **p < 0.01; ***p < 0.001.

During tumor progression, the spatiotemporal interactions between tumor and immune cells build an ITM, enabling tumor cells to escape immune attack. Especially, IDO‐1 is highly expressed in cancer and immune cells, which severely impairs immune responses through the depletion of Trp and the accumulation of Kyn. Trp depletion inhibits the survival and function of effector T cells, and Kyn accumulation promotes the generation of Tregs. Immunofluorescence staining revealed that HMGB1 efflux and CRT exposure in tumor tissue were amplified by SNPs (Figure S26, Supporting Information). These ICD‐related DAMPs, can cascadingly bind to pattern recognition receptors on DCs, promote DCs maturation, strengthen antigen present, and activate T cells. We then studied DCs maturation in tumor‐draining lymph nodes (TDLNs) by flow cytometry analysis. The high expression of CD80^+^CD86^+^ on DCs after SNPs treatment demonstrated that this therapeutic modality promoted DCs maturation (**Figure** **5**a,b), which strengthened the tumor immunogenicity. Immunohistochemical staining and flow cytometry were used to evaluate T lymphocyte differentiation and infiltration. As shown in Figures S27–S30 (Supporting Information), the proportion of T lymphocytes in tumor tissue was raised for the mice treated with SNPs. Furthermore, the percentages of infiltrating CD4^+^ and CD8^+^ T cells in the spleen were measured to be 7.75% (Figure 5c,d) and 12.0% (Figure 5e,f), respectively, which were much higher than those in other groups. Compared with CD4^+^ helper T cells, the homing and trafficking of CD8^+^ cytotoxic T cells was more effective (Figure S31, Supporting Information). CD4^+^ helper T cells not only differentiate into effective T cells (CD3^+^CD4^+^Foxp3^−^) but also immunosuppressive Tregs (CD3^+^CD4^+^Foxp3^+^). The population of Tregs in the SNPs group decreased by 65% compared with the PBS group (Figure 5g,h), and also much lower than those in IDOi, DACHPt, and IDOi@DACHPt groups, suggesting SNPs effectively reprogramed ITM.

The activation of systematic antitumor immune responses was explored by determining the intratumoral and hematological secretion of immunocompetent cytokines using enzyme‐linked immunosorbent assays (ELISA). The sera and tumor tissues were collected on the 1, 3, and 7 d after last administration. Figure 5i revealed that SNPs treatment not only upregulated the level of proinflammatory cytokines including interleukin‐6 (IL‐6), IFN‐γ and tumor necrosis factor‐α (TNF‐α) but also prolonged their period of validity, suggesting SNPs efficiently boosted the antitumor immune responses. IFN‐𝛾 not only serves as a cytotoxic cytokine to build a positive feedback loop but also stimulates other immunosuppressive mechanisms via upregulating the IDO pathway (Figure S32, Supporting Information). The IDO‐1 activity was then assessed by determining the intratumoral metabolism of Trp and Kyn. The lowest Kyn/Trp ratio was detected in the SNPs group, which was 10.4‐fold lower than that of the PBS group due to IDOi‐mediated IDO‐1 inhibition (Figure 5k).

The population of cytotoxic T cells in the SNPs group kept high even on 14 d post‐last administration (Figure S33, Supporting Information), which demonstrated that SNPs guaranteed long‐term antitumor immunity. To prove SNPs‐induced immune responses for the prevention of tumor recurrence and metastasis, a rechallenging tumor model was constructed by i.v. injecting CT26 cells after chemoimmunotherapy. As displayed in Figure 5j,l a mass of pulmonary metastatic nodules were found in the lung tissues of mice treated with PBS, IDOi, and IDOi@DACHPt, but no tumor node metastasis was observed for the mice treated with SNPs, verifying the potential of SNPs for the long‐term suppression of tumor metastasis. This observation was further verified by lung H&E tissue sections. As displayed in Figure 5m, obvious massive tumor metastasis nodules were observed in the lung tissues of the mice treated with PBS and IDOi, and the lung metastasis was suppressed at various levels by DACHPt and IDOi@DACHPt treatments. Impressively, no tumor metastasis nodules were observed in the SNPs group.

## 3 Conclusion

In summary, we developed a supramolecular modulation of tumor microenvironment to amplify tumor chemoimmunotherapy. DACHPt was efficiently loaded into SNPs to implement chemotherapy and activate the antitumor immune response with the help of metal coordination. Meanwhile, the host–guest complexation‐assisted IDOi encapsulation suppressed the catabolism of Trp and Kyn, synergistically reversing the “cold” tumor into a “hot” tumor to potentiate chemoimmunotherapy. This supramolecular combination of IDOi and DACHPt robustly remodeled the ITM and achieved remarkable antitumor efficacy in vitro and in vivo, which was supported by the maturation of DCs and the renaissance of T lymphocyte recruitment in tumor sites. Importantly, long‐term antitumor immune responses were evoked by SNPs, effectively improving the prognosis of the tumor. This supramolecular modulation strategy provides a blueprint to direct the design of the next generation of supramolecular nanomedicines with excellent patients' prognoses.

## 4 Experimental Section

### Ethics Statements for Animal Experiments

All animal experiments were carried out following the guidelines set by the Ethics Committee of Zhejiang University of Technology. The study protocol received approval from the Zhejiang University of Technology Animal Care and Use Committee (20230704040).

### Statistical Analysis

All experiments were investigated at least three times, and the data were shown as mean ± standard deviation. Statistical significance was calculated via ordinary one‐way ANOVA with a Tukey's test. *p < 0.05; **p < 0.01; ***p < 0.001.

## Conflict of Interest

The authors declare no conflict of interest.

## Supporting information



Supporting Information

## Data Availability

The data that support the findings of this study are available from the corresponding author upon reasonable request
