# Supplementary material for: Supramolecular Modulation of Tumor Microenvironment Through Host−Guest Recognition and Metal Coordination to Potentiate Cancer Chemoimmunotherapy
Source: Adv Sci (Weinh). 2025 Jan 30;12(11):2408518. doi: 10.1002/advs.202408518 (PMC11923969; doi:10.1002/advs.202408518)
Supplement: Supplementary file 1 — Supporting Information [file ADVS-12-2408518-s001.docx]

Supporting Information

Supramolecular Modulation of Tumor Microenvironment through Host−Guest Recognition and Metal Coordination to Potentiate Cancer Chemoimmunotherapy

Dan Wu,^*^ Jie Zhou, Zhankui Zhang, Yibin Cao, Kunmin Ping, Shaolong Qi, Jianshi Du,^*^ and Guocan Yu^*^

Prof. Dr. D. Wu, J. Zhou, Z. Zhang, Y. Cao, K. Ping

College of Materials Science and Engineering, Zhejiang University of Technology, Hangzhou, 310014, P. R. China.

E-mail: [danwu@zjut.edu.cn](mailto:danwu@zjut.edu.cn)

Dr. S. Qi, Prof. Dr. J. Du

Vascular Surgery Center, the Third Hospital of Jilin University, Changchun 130031, P. R. China

E-mail: [dujs@jlu.edu.cn](mailto:dujs@jlu.edu.cn)

Dr. S. Qi, Prof. Dr. G. Yu

Ministry of Education Key Laboratory of Bioorganic Phosphorus Chemistry & Chemical Biology, Department of Chemistry, Tsinghua University, Beijing 100084, P. R. China.

E-mail: [guocanyu@mail.tsinghua.edu.cn](mailto:guocanyu@mail.tsinghua.edu.cn)

1. **Materials and method**

**Materials**

All reagents were commercially available and used as supplied without further purification. Solvents were either employed as purchased or dried according to procedures described in the literature. PEG-BLG-CD and IDOi were synthesized according to literature procedures. ^1^H NMR spectra were recorded on a Bruker Avance DMX 400 spectrometer. 2D NOESY spectra were recorded on a Bruker Avance Ⅲ-600 spectrometry. Fluorescence spectra were taken on a F-4600 FL spectrophotometer 2314-016. UV-vis spectra were taken on a UV-1900 spectrophotometer (Shimadzu, Japan). Isothermal titration calorimetry (ITC) was titrated on a VP-ITC (Malvern, Britain). Hydrodynamic size and zeta potential were measured on a Nano Brook Omni. X-ray photoelectron spectroscopy (XPS) was measured on a ESCALAB220i-XL (VG Scientific, Britain). Transmission electron microscopy (TEM) images were taken on a 120 kv HT7800 transmission electron microscope (Hitachi, Japan). The absorbance of formazan at 490 nm in MTT experiment was measured using a BIO-RAD microplate (IMark, Germany). High performance liquid chromatography (HPLC) was performed on an Agilent 1260 Infinity II. Inductively coupled plasma mass spectrometry (ICP-MS) was measured on an ICP-9800 (Shimadzu, Japan). Flow cytometry was performed on a BD FACSCanto II flow cytometer (Becton, America) and the data was analyzed using Flowjo software. Confocal laser scanning microscopy (CLSM) images were taken on a NCF950 confocal laser scanning microscope (Ningbo Yongxin Optics, China). The fluorescence photomicrographs were taken using a TS2-FL Fluorescent Inverted microscope Eclipse (Nikon, Japan). *In vivo* fluorescence imaging was conducted on an IVIS small-animal spectrum (PerkinElmer, America).

**Methods**

**Preparation of SNPs**

First, 40.0 mg of PEG-BLG-CD polymer was dissolved in 2.0 mL of DMF and sonicated for 1 h. Then DACHPt (6.00 mg) dissolved in 0.5 mL of DMF and IDOi (7.00 mg) dissolved in 0.5 mL of DMF were added into the PEG-BLG-CD solution. The mixture was slowly dropped into 5.0 mL of deionized water, followed by 60 min ultrasound treatment, and eventually stirred at 50 ^o^C for 48 h. SNPs was obtained through vacuum freeze drying.

**Host**−**guest complexation between *β*-CD and IDOi**

Solutions of IDOi (1.00 mM), IDOi (1.00 mM) + *β*-CD (1.00 mM) and *β*-CD (1.00 mM) in D_2_O were prepared, respectively, and their ^1^H NMR spectra were measured on the Bruker AVANCE DMX 400 spectrometer.

**Morphological characterization of SNPs**

The hydrodynamic particle size and surface charge of SNPs were determined using dynamic light scattering, and the morphology of SNPs was examined using transmission electron microscope.

**Drug release profile of SNPs**

1.0 mL of SNPs aqueous solution (4.30 mM) was added into dialysis bag (1 kDa MWCO), which was immersed into a 50 mL centrifuge tube containing 39.0 mL of PBS buffer (*v*_DMF_/*v*_PBS_ = 3/7), and then oscillated at 37.0 °C. 1.0 mL of dialysate was taken out at the determined time points for UV measurement. At the same time, 1.0 mL of fresh PBS buffer (*v*_DMF_/*v*_PBS_ = 3/7) was added back into dialysate. The release profile of DACHPt was monitored by ICP-MS, and the release profile of IDOi was supervised by HPLC, in which the mixture of acetonitrile and water (*v*/*v* = 90/10) served as mobile phase.

**Incorporation of BODIPY in SNPs**

DACHPt (6.00 mg), BODIPY containing a carboxylate group (1.00 mg) and PEG-BLG-CD (40.0 mg) were first dissolved in 2 mL of DMF, followed by 60 min ultrasound treatment. Then, the DMF solution dissolved with 7.0 mg of IDOi was gradually added into the above mixture solution, which was subsequently added into 5 mL of deionized water and further sonicated for 60 min. The mixture was further stirred at 50 ^o^C for 24 h, and the free components were eliminated through dialysis.

**Cell uptake of SNPs**

First, CT26 cells were seeded in 24-well plates at a density of 5 × 10^4^ cells/well, and allowed to adhere overnight. Then BODIPY-labeled SNPs (11.0 μM) was cultured with CT26 cells for 2, 4 and 8 h, respectively, followed by 15 min DAPI staining. Eventually, CT26 cells were washed with PBS and fixed with fresh 4.0% paraformaldehyde for 15 min. The CLSM images were taken using a confocal laser scanning microscope.

**Evaluation of cytotoxicity**

First, CT26 cells were seeded in 96-well plates at a density of 1 × 10^4^ cells/well, and allowed to adhere overnight. Then different formulations were cultured with CT26 cells for 24 h. The medium was removed and 100 μL of fresh MTT solution (0.50 mg/mL) was added to each well. After incubation for 4 h, MTT solution was removed and 150 μL of DMSO was added to each well. Eventually, the UV absorbance of the formazan product at 490 nm was measured using a microplate reader.

**Live/dead dual-staining**

First, CT26 cells were seeded in 96-well plates at a density of 1 × 10^4^ cells/well, and allowed to adhere overnight. Then different formulations were cultured with CT26 cells for 12 h. Cells were washed with PBS and stained with calcein acetoxymethyl ester (2.0 μM) and propidium iodide (5.0 μM) in the dark for 20 min at 37°C. The fluorescence images were taken on a fluorescent inverted microscope.

**Apoptosis induction**

First, CT26 cells were seeded in 96-well plates at a density of 1 × 10^4^ cells/well, and allowed to adhere overnight. Then different formulations were cultured with CT26 cells for 12 h. Cells were washed with PBS and stained with fluorescein isothiocyanate (2.0 μL) and propidium iodide (5.0 μL) in the dark for 20 min at 37 °C. The programmed death manner of cells was analyzed using a flow cytometer.

**Immunogenic cell death of CT26 cells**

CT26 cells were seeded in 96-well plates at a density of 1 × 10^4^ cells/well, and allowed to adhere overnight. Then different formulations were cultured with CT26 cells for 12 h. Cells were fixed with 4% paraformaldehyde, and stained with HMGB1 primary antibody for 1 h, goat Alexa Fluor 488 anti-Mouse IgG secondary antibody for 1 h and DAPI staining for 5 min, respectively. The fluorescence images were taken on a fluorescent inverted microscope. For CRT staining, similar method was used. Extracellular ATP content was measured according to the standard protocol.

**IDO-1 pathway inhibition**

CT26 cells were seeded in 96-well plates at a density of 1 × 10^4^ cells/well, and allowed to adhere overnight. Then different formulations were cultured with CT26 cells for 12 h. Recombinant mouse IFN-γ (100 ng/mL) was added into each well and incubated with cells for 24 h. The supernatant of each well was transferred to a new 96-well plate, and the contents of Trp were measured using HPLC in which the mixture of acetonitrile/water (*v*/*v*=8/92, pH = 3.6) served as mobile phase, while the contents of Trp were measured using a microplate reader (490 nm).

**Pharmacokinetic study**

Animal care and handling procedures were in agreement with the guidelines evaluated and approved by the ethics committee of Zhejiang University of Technology. Study protocols (ethic code: 20230704040) involving animals were approved by the Zhejiang University of Technology Animal Care and Use Committee. 8 healthy SD mice (7 weeks) were first randomly divided into 4 groups. Then, the mice were *i.v.* injected with IDOi (2.00 mg/kg), DACHPt (2.30 mg/kg) and SNPs (IDOi: 2.00 mg/kg, DACHPt: 2.30 mg/kg), respectively. At the determined time points, blood samples (300 μL) were collected from orbit, and then centrifuged for 5 min (4000 rpm). DACHPt content was measured using ICP-MS and IDOi content was determined by HPLC.

***In vivo* fluorescence imaging**

CT26 tumor-bearing mice were *i.v.* administered with 200 μL of SNPs solution (1.0 mg kg^-1^). At the determined time points, mice were anesthetized and imaged by an *in vivo* imaging system (λ_ex_ = 710 nm, λ_em_ = 760 nm). After *in vivo* imaging, mice were euthanized, and main organs and tumor tissues were collected for *ex vivo* imaging.

**Antitumor therapy**

BALB/c mice (7 weeks) were purchased from Hangzhou Keling Biotechnology Co., Ltd and used under the approved protocols (ethic code: 20230704040) of the animal experimental center of Zhejiang university of technology. 1 × 10^6^ CT26 cells suspended in 100 μL of PBS were subcutaneously implanted into the left flank of mice. The mice were used in experiments when the tumor volumes reached 100 mm^3^. CT26 tumor-bearing mice were randomly divided into 5 groups (*n* = 6). Mice were *i.v.* administered with 100 μL of PBS, IDOi (10.0 mg kg^-1^), DACHPt (5.00 mg kg^-1^), IDOi@DACHPt (35.0 mg kg^-1^) and SNPs (35.0 mg kg^-1^) every 3 days. The tumor size was measured every 3 days and the tumor volume *V* was calculated according to the formula *V* = (tumor length) × (tumor width)^2^/2. After treatments, mice were euthanized, and main organs (heart, liver, spleen, lung, kidney, tumor-draining lymph node) and tumor tissues were collected. On day 12, 100 μL of PBS containing 3 × 10^5^ CT26 cells was *i.v.* injected to establish rechallenging tumor model. On day 24, all mice were euthanized, and the number of metastatic nodules in the lung tissues was counted and the lung tissues were stained with hematoxylin and eosin (H&E). Tumors were homogenized in PBS to obtain single-cell suspension, and the cell suspensions were stained by anti-CD3ε-PE, anti-CD4-FITC, anti-CD8a-PE-Cy7, anti-CD25-APC and anti-FoxP3-PE and analyzed by flow cytometry. The lymph nodes of mice were gathered, homogenized in PBS, and filtered to gain single-cell suspension, and the cell suspension of lymph nodes was stained with anti-CD11c-FITC, anti CD80-PE and anti-CD86-APC antibodies and analyzed by flow cytometry. During treatment, blood samples of mice at day 1, 3 and 7 were collected, and the level of TNF-α, INF-γ and IL-6 was detected by ELISA Kit.

1. **Synthesis of *β*-CD-NH_2_**

**Synthesis of *β*-CD-OTs**

*β*-CD (20.0 g, 17.6 mmol) was first suspended in 167 mL of water, then NaOH (2.19 g, 54.7 mmol) dissolved in 7.0 mL of water was added dropwise. The mixture was immersed into the ice-water bath, and TsCl (5.04 g, 26.4 mmol) dissolved in 10 mL of acetonitrile was dripped slowly. After 2 h, the mixture was refrigerated overnight at 4 ^o^C. The resulting precipitate was collected *via* suction filtration and recrystallized in hot water for three times. ^1^H NMR (400 MHz, DMSO-*d*_6_) δ 7.77–7.75 (d, 2H), 7.45–7.43 (d, 2H), 5.74 (m, 14H), 4.84–4.78 (m, 6H), 4.52 (m, 6H), 4.20 (m, 1H), 3.66–3.20 (m, 42H), 2.44 (s, 3H).

**Figure S1.** ^1^H NMR spectrum of *β*-CD-OTs.

**Synthesis of *β*-CD-NH_2_**

*β*-CD-OTs (5.00 g, 3.88 mmol) was dissolved in 30 mL ethylenediamine, and stirred at 70 ^o^C. After 24 h, the mixture was cooled down and poured into acetone (100 mL). The precipitate was collected *via* centrifugation and washed with acetone for three times. ^1^H NMR (400 MHz, DMSO-*d*_6_) δ 5.73 (m, 14H), 4.91–4.76 (m, 7H), 4.75–4.17 (m, 6H), 3.79–3.45 (m, 28H), 3.33–3.28 (m, 12H), 3.17 (s, 1H), 3.00–2.63 (m, 5H).

**Figure S2.** ^1^H NMR spectrum of *β*-CD-NH_2_.

1. **Synthesis of PEG-BLG-CD**

**Synthesis of PEG-BLG**

**
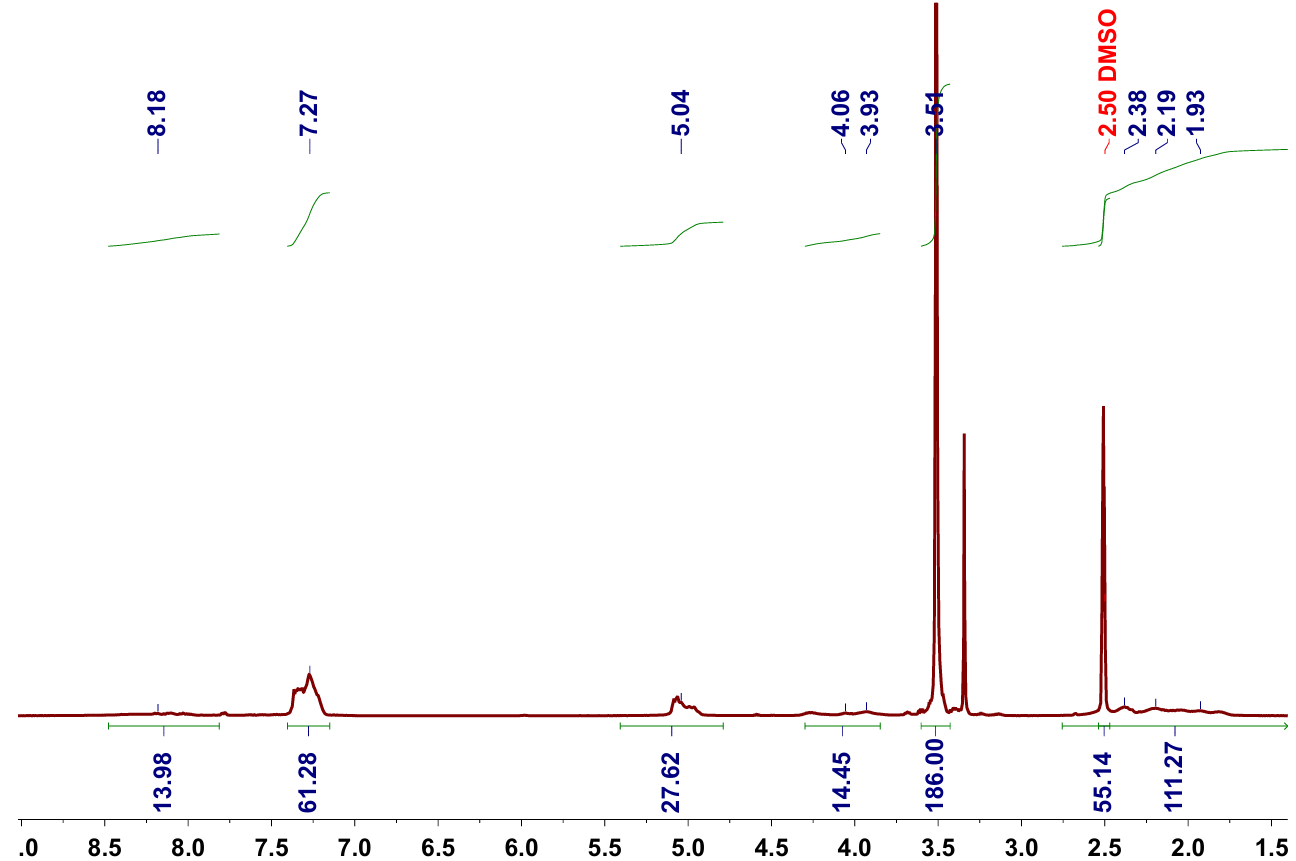
**

**Figure S3.** ^1^H NMR spectrum of PEG-BLG.

γ-Benzyl-L-glutamate-*N*-carboxyanhydride (0.24 g, 1.00 mmol) was dissolved in anhydrous THF (20 mL). Then a 0.1 M LiHMDS in THF (6 mL) was added slowly. The mixture was stirred for 30 min at room temperature, then was added with MeO-PEG-NH_2_ (0.20 g, 0.10 mmol). The polymerization was quenched by a few drops of HCOOH. The resulting solution was poured into cold acetone (400 mL) and the precipitation was collected *via* centrifugation. The collected coarse product was dissolved in THF (15 mL) and poured into ethyl ether (400 mL). The precipitate was collected *via* centrifugation and washed with ethyl ether for three times. ^1^H NMR (400 MHz, DMSO-*d*_6_)) δ 8.18 (m, 14H), 7.27 (m, 61H), 5.04 (m, 28H), 4.06–3.93 (m, 14H), 3.51 (m, 186H), 2.38–1.93 (m, 56H).

**Synthesis of PEG-BLG-COOH**

PEG-BLG (5.00 g) was first dissolved in 50 mL of dichloroacetic acid at 25 ^o^C. Then 15.0 mL of hydrobromic acid/acetic acid (33 wt%) was slowly added and the mixture was stirred at 30 ^o^C for 1 h. The final product was poured into excess diethyl ether and the precipitate was collected *via* centrifugation. ^1^H NMR (400 MHz, DMSO-*d*_6_) δ 8.29 (m, 15H), 7.28 (m, 29H), 5.04 (m, 12H), 3.94 (m, 16H), 33.51 (m, 186H), 3.33 (m, 18H), 2.24–1.99 (m, 57H).


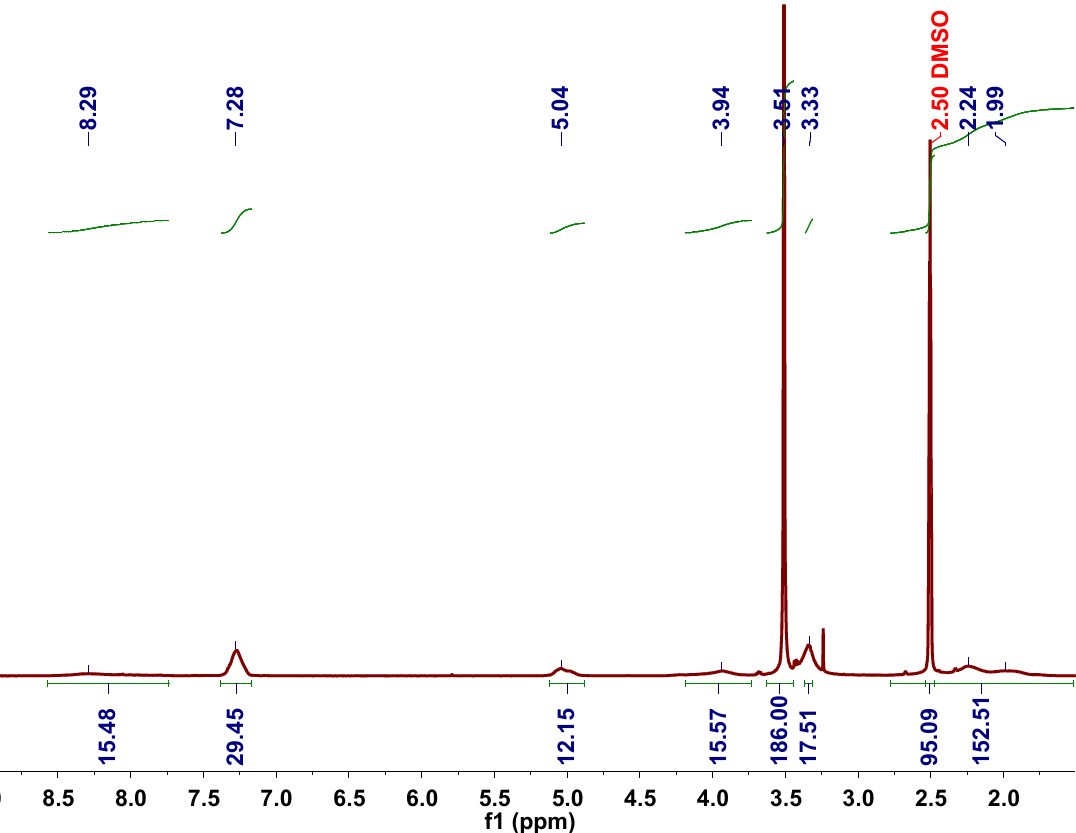


**Figure S4.** ^1^H NMR spectrum of PEG-BLG-COOH.

**Synthesis of PEG-BLG-CD**

The polymer PEG-BLG-COOH (1.00 g, 0.30 mmol) was first dissolved in 10 mL of DMF, then [*N*-hydroxysuccinimide](https://www.chemsrc.com/en/cas/6066-82-6_671474.html) (NHS) (136 mg, 1.00 mmol), [1-(3-dimethylaminopropyl)-3-ethylcarbodiimide hydrochloride](https://www.chemsrc.com/en/cas/25952-53-8_1089456.html) (EDC) (200 mg, 1.00 mmol) and dimethylaminopyridine (DMAP) (10.0 mg, 0.08 mmol) were added. After 20 h, the mixture was dropped into excess diethyl ether and the precipitate was collected *via* centrifugation. The carboxyl-activated polymer and *β*-CD-NH_2_ (470 mg, 0.40 mmol) were dissolved in 10 mL of DMF, and 20 μL of triethylamine was added. After 24 h, The final product was dropped into excess diethyl ether and the precipitate was collected *via* centrifugation. ^1^H NMR (400 MHz, DMSO-*d*_6_)

δ 8.21 (m, 12H), 5.77–5.70 (m, 82H), 4.84 (m, 41H), 4.52–4.49 (m, 42H), 4.13–3.97 (m, 12H), 3.91–3.58 (m, 163H), 3.57–3.41 (m, 186H), 3.34–3.22 (m, 71H), 2.11–1.83 (m, 48H).


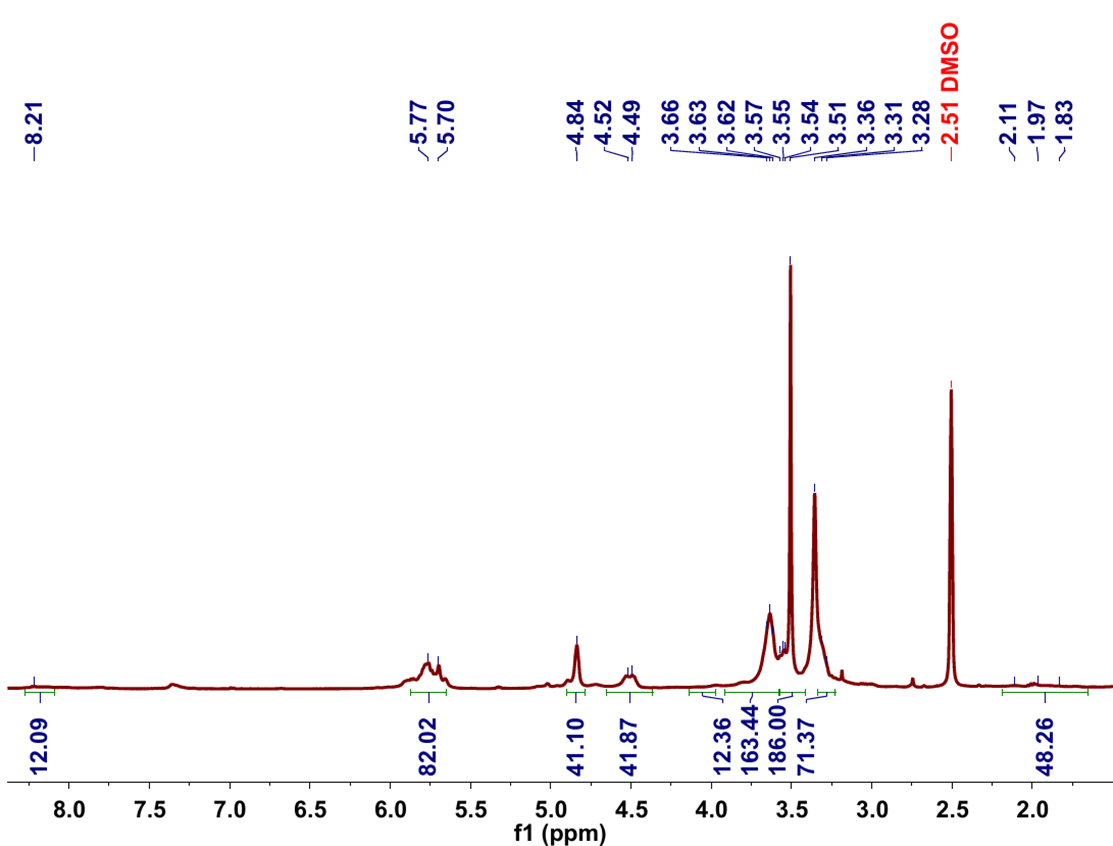


**Figure S5.** ^1^H NMR spectrum of PEG-BLG-CD.

**Figure S6.** The average molecular weight (*M*_n_) of PEG-BLG-CD measured by GPC.

1. **Structural characterization of PEG-BLG-CD**

**Figure S7.** The illustration of metal coordination between DACHPt and IDOi.


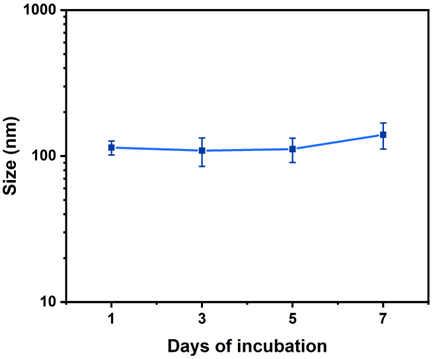


**Figure S8.** The average diameter change of SNPs during a 7-day incubation in PBS.


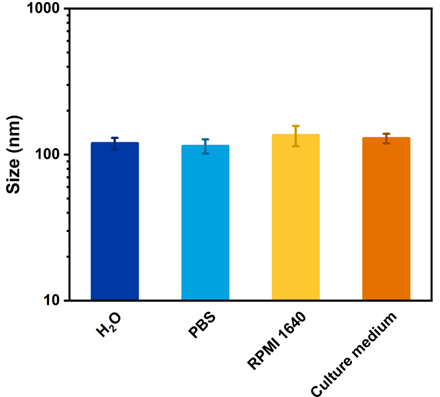


**Figure S9.** The average diameter of SNPs after incubation in H_2_O, PBS, RPMI 1640 and culture medium for 24 h.


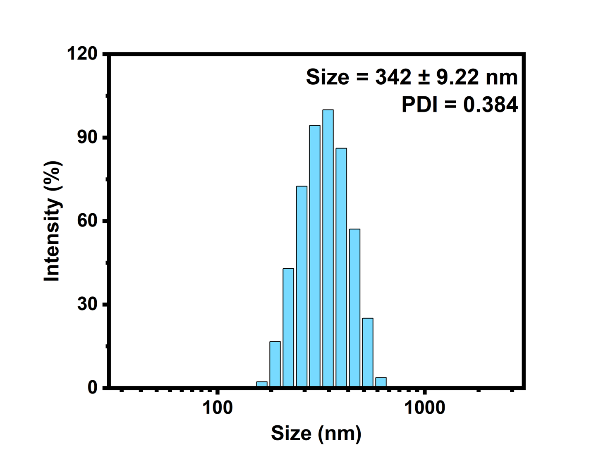


**Figure S10.** DLS result of SNPs at pH 5.5.

1. ***In vitro* study of SNPs**


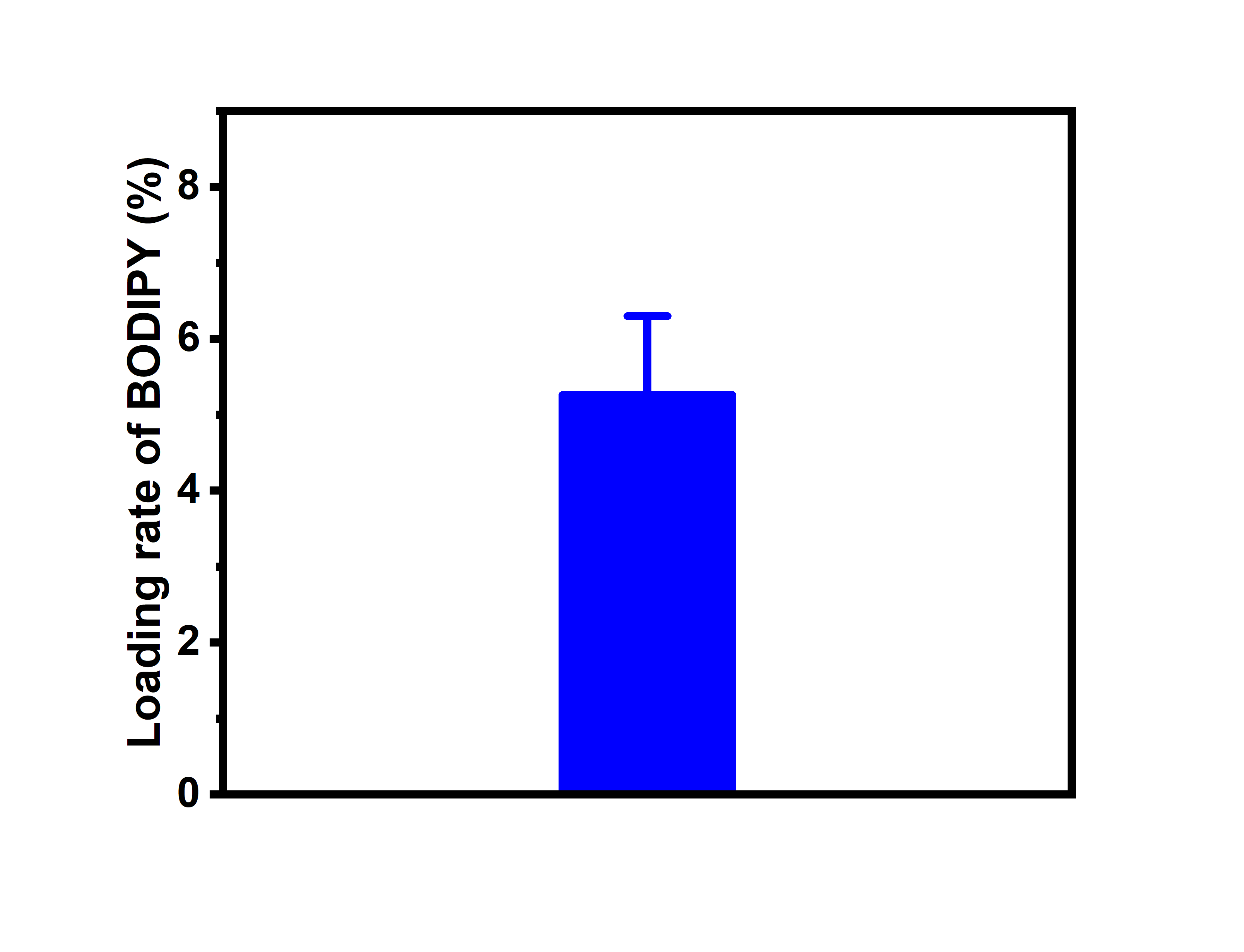


**Figure 11.** The loading content of BODIPY in SNPs.


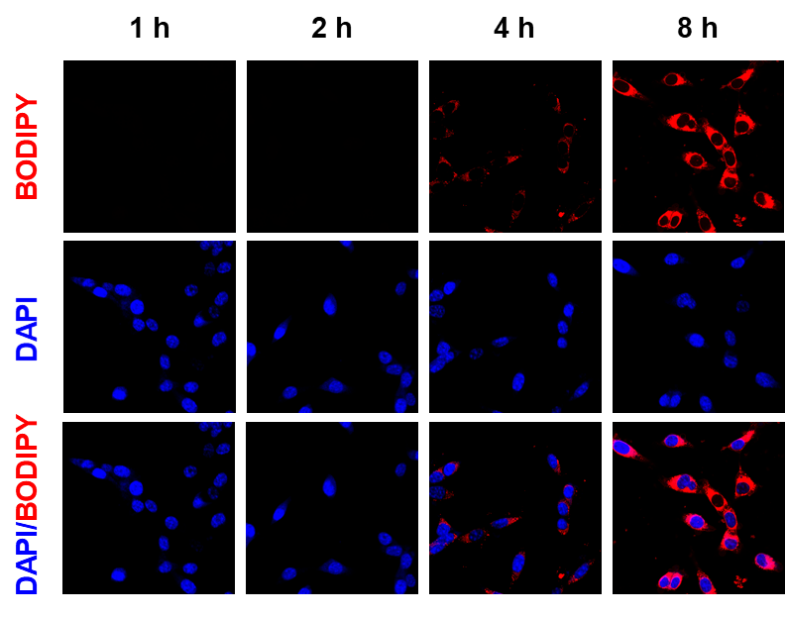


**Figure S12.** CLSM images of CT26 cells treated with BODIPY-labeled SNPs for 1 h, 2 h, 4 h and 8 h.


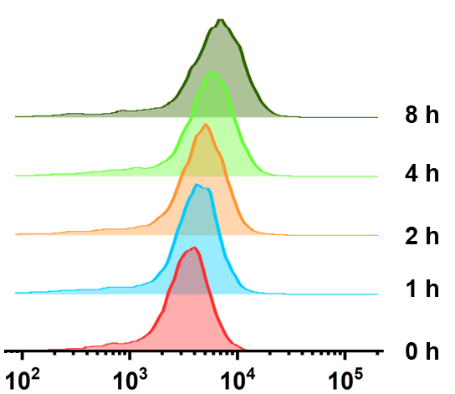


**Figure S13.** Flow cytometry analysis of the endocytosis of SNPs.


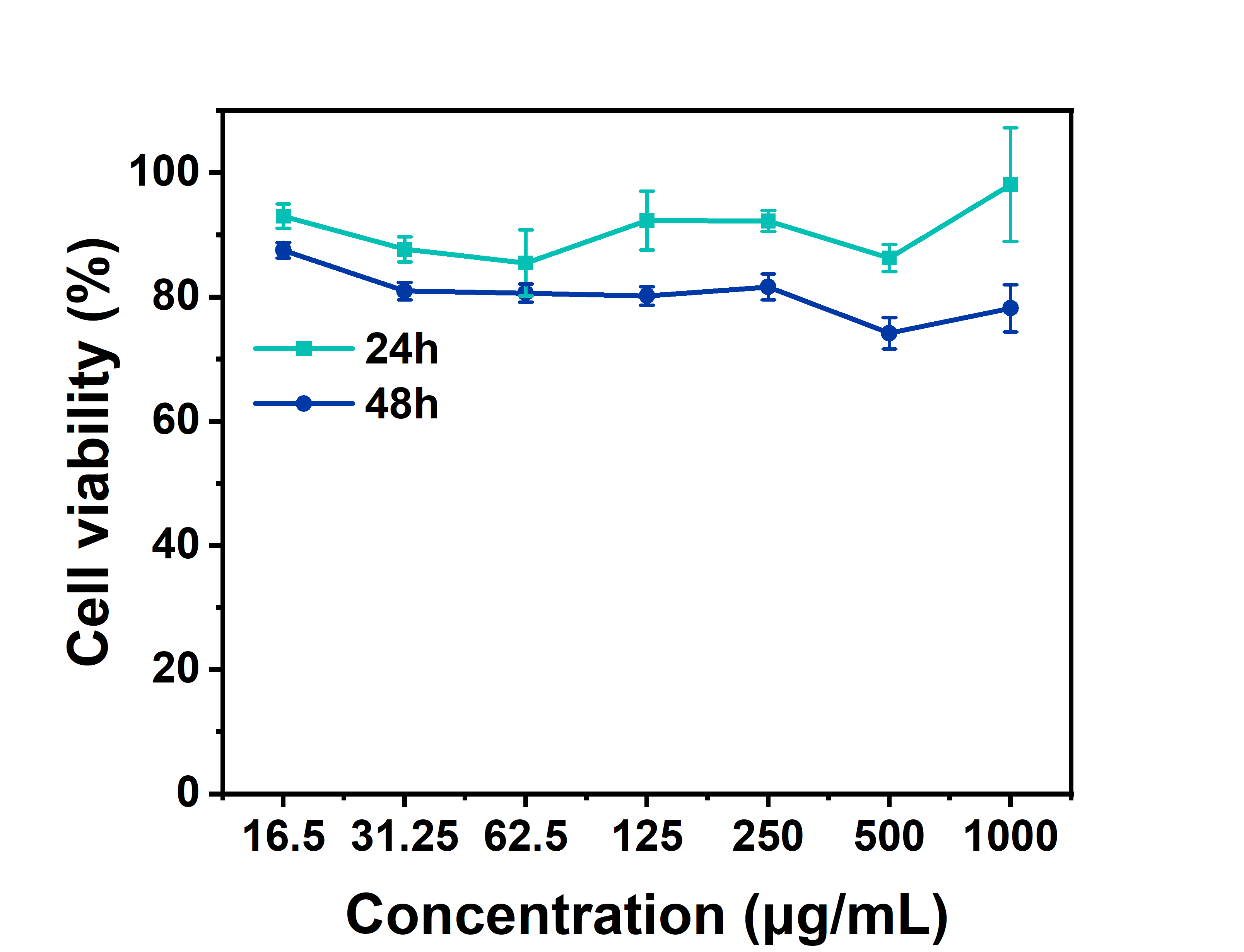


**Figure S14.** Cell viability of CT26 cells treated with different concentrations of PEG-BLG-CD.


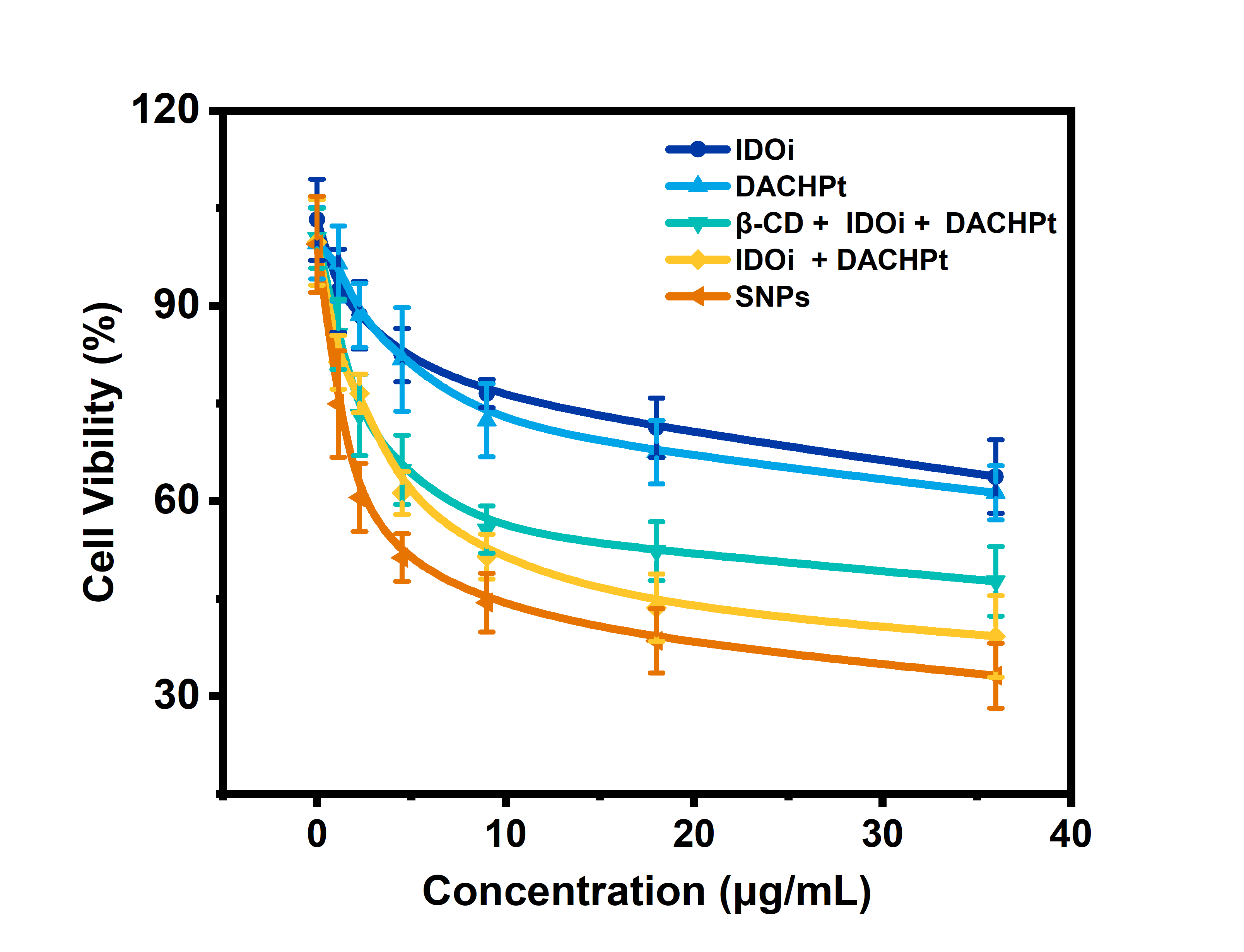


**Figure S15.** Cell viability of 4T1 cells treated with different formulations.


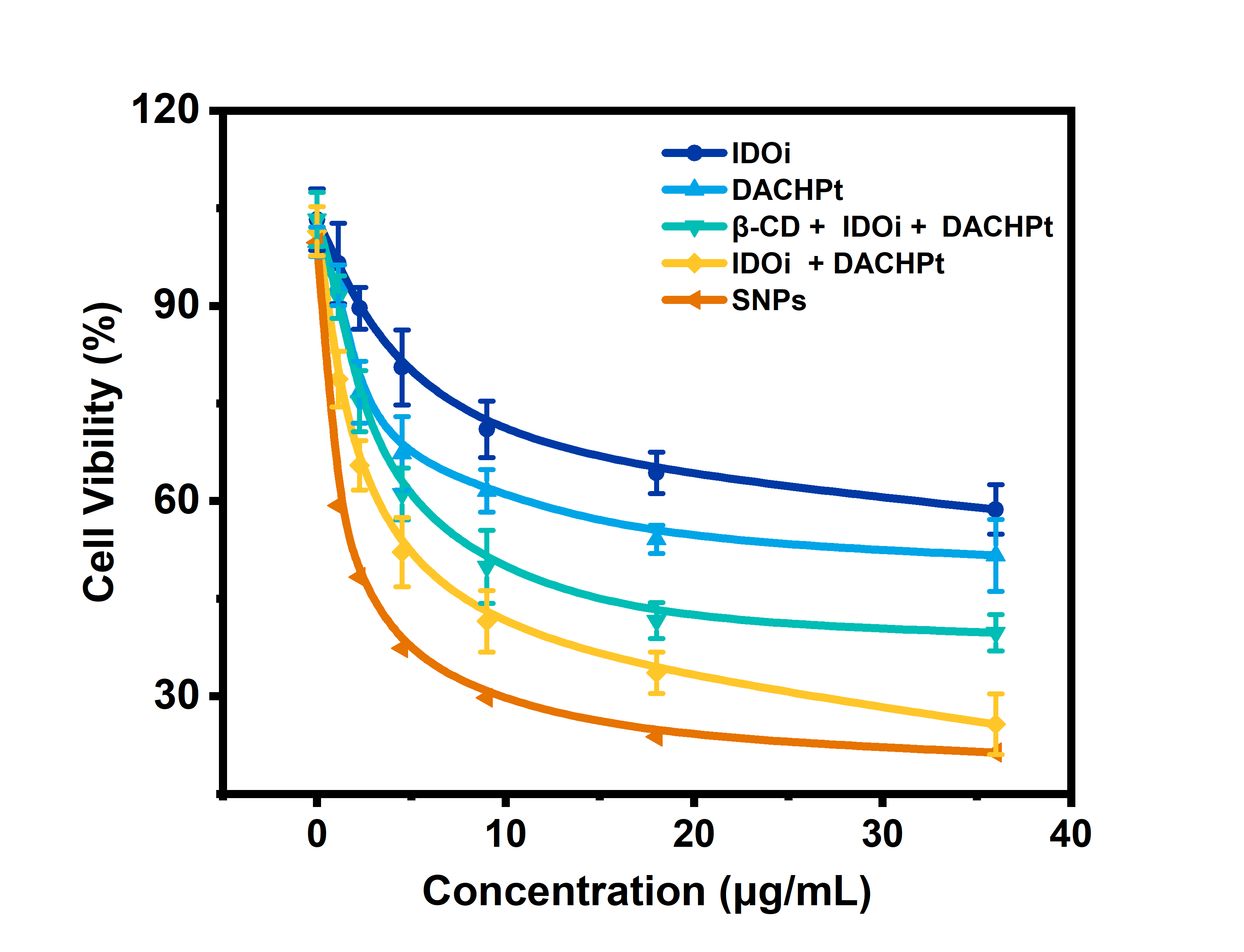


**Figure S16.** Cell viability of HeLa cells treated with different formulations.


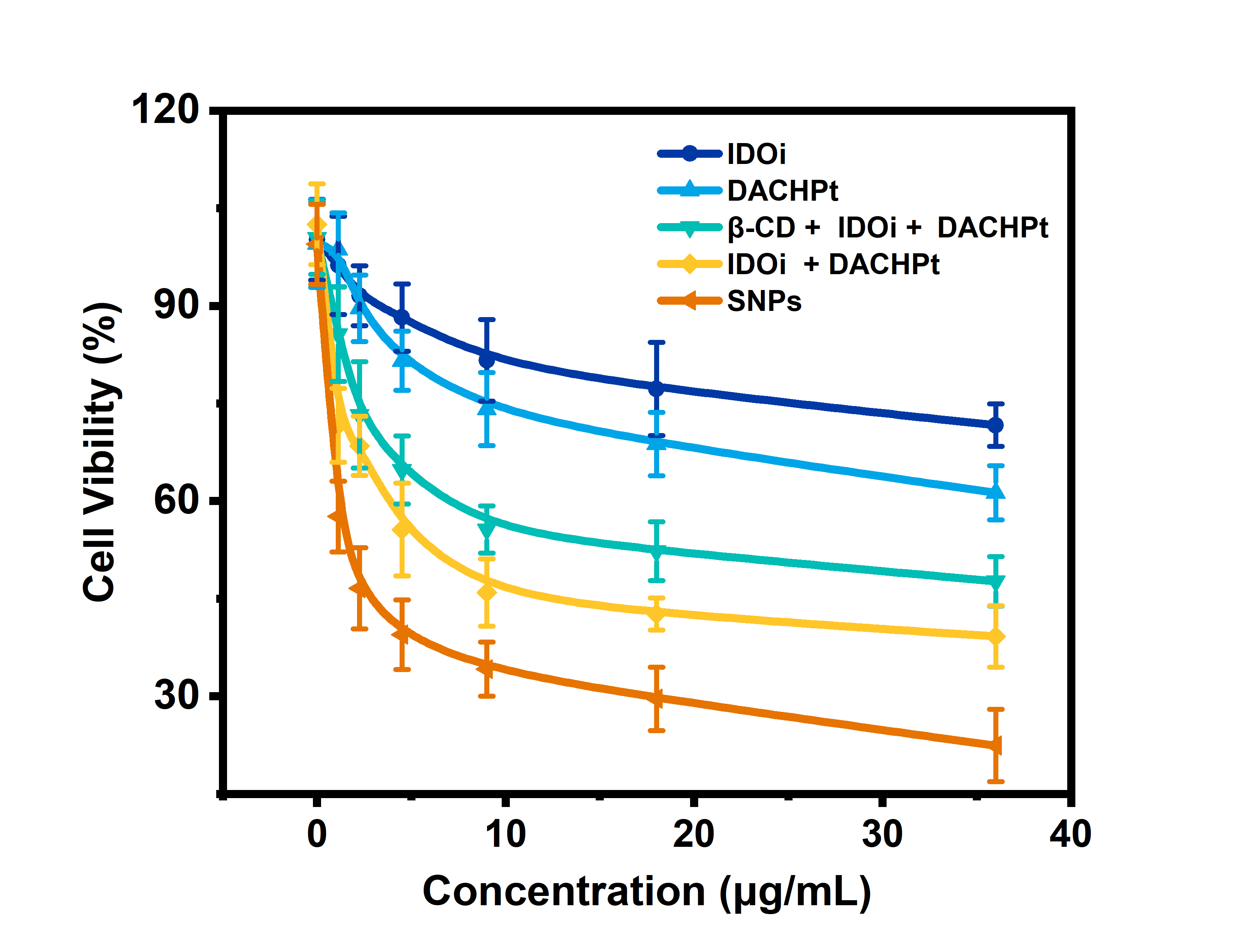


**Figure S17.** Cell viability of A549 cells treated with different formulations.


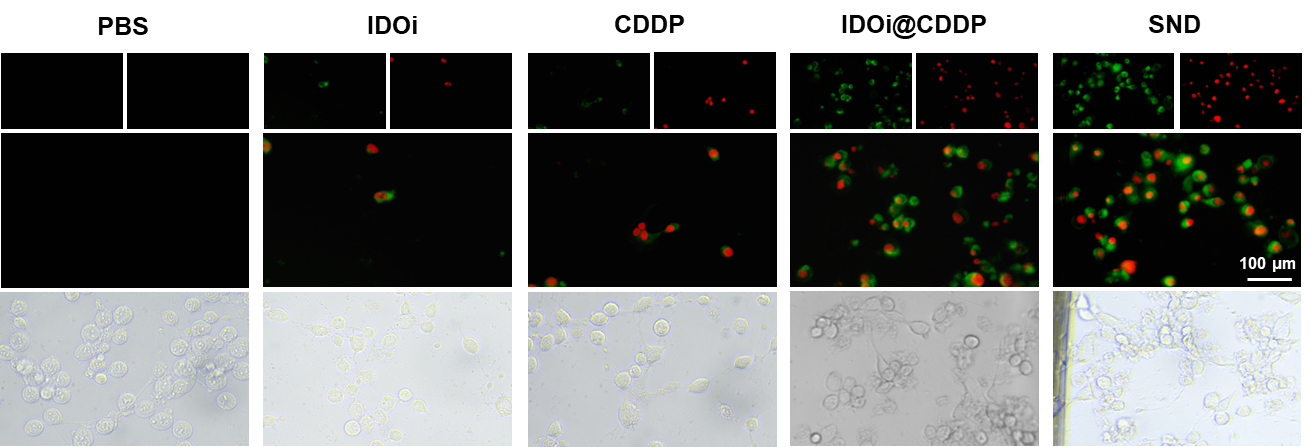


**Figure S18.** Annexin-V-FITC/PI staining fluorescence images of CT26 cells after different treatments.


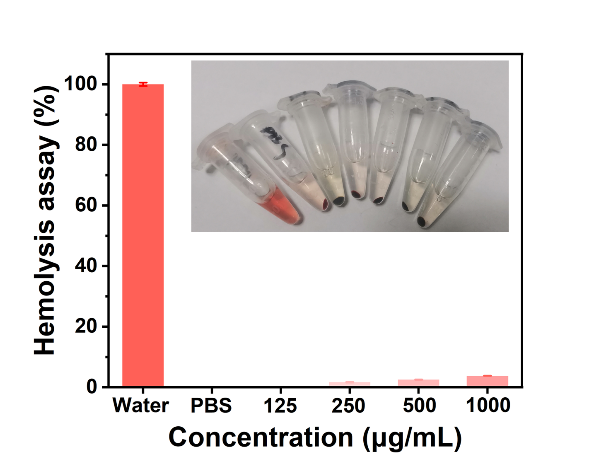


**Figure S19.** Haemolysis rates of SNPs at various concentrations.
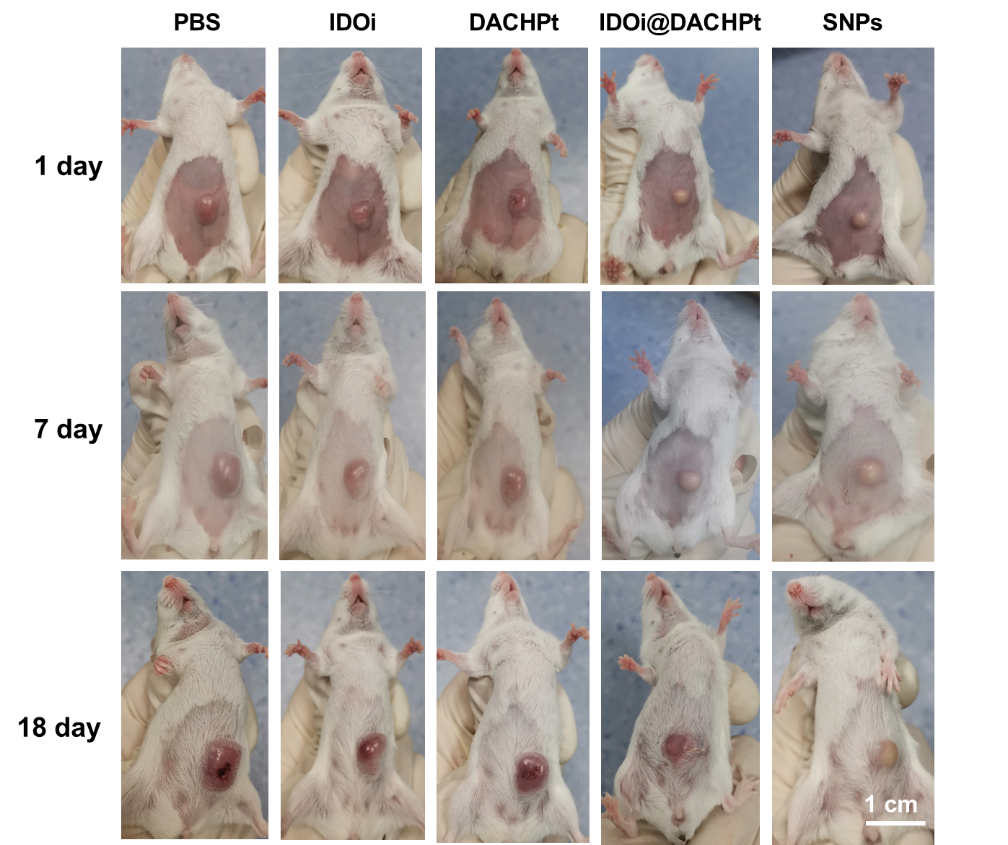


**Figure S20.** The pictures of mice bearing CT26 tumor during different treatments.


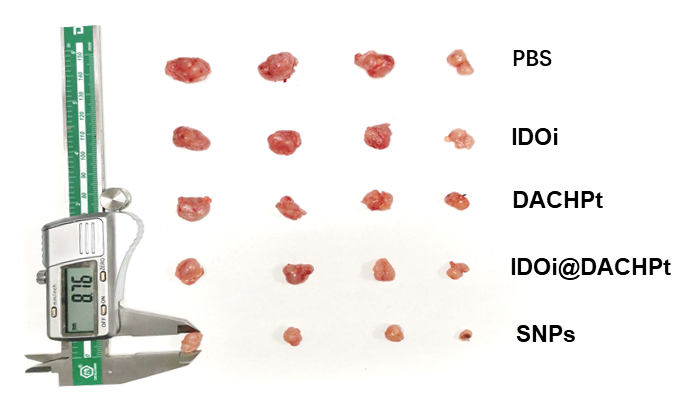


**Figure S21.** The pictures of tumor tissues of mice after different treatments.

1. ***In vivo* study of SNPs**


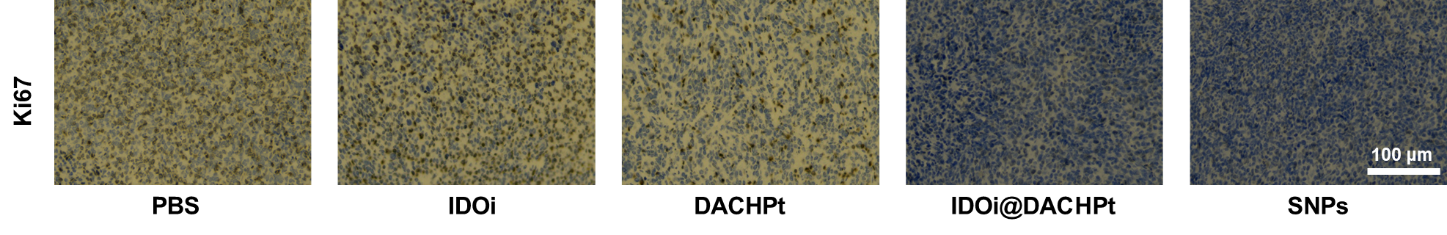


**Figure S22.** Ki67 staining of tumor tissues of mice after different treatments.


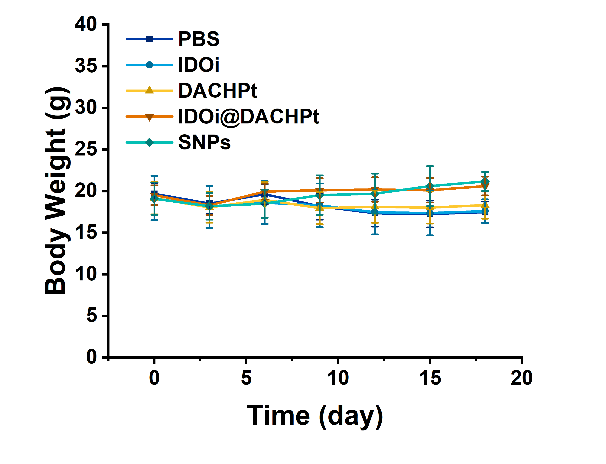


**Figure S23.** Average body weights of mice after different treatments.


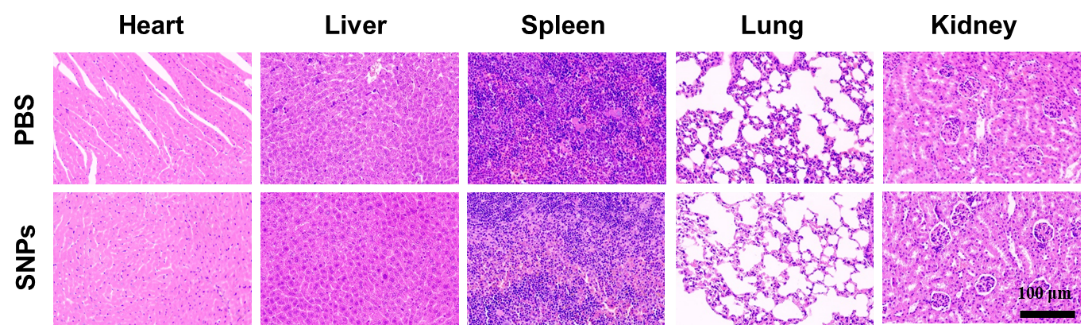


**Figure S24.** H&E staining of main organs of mice after different treatments.


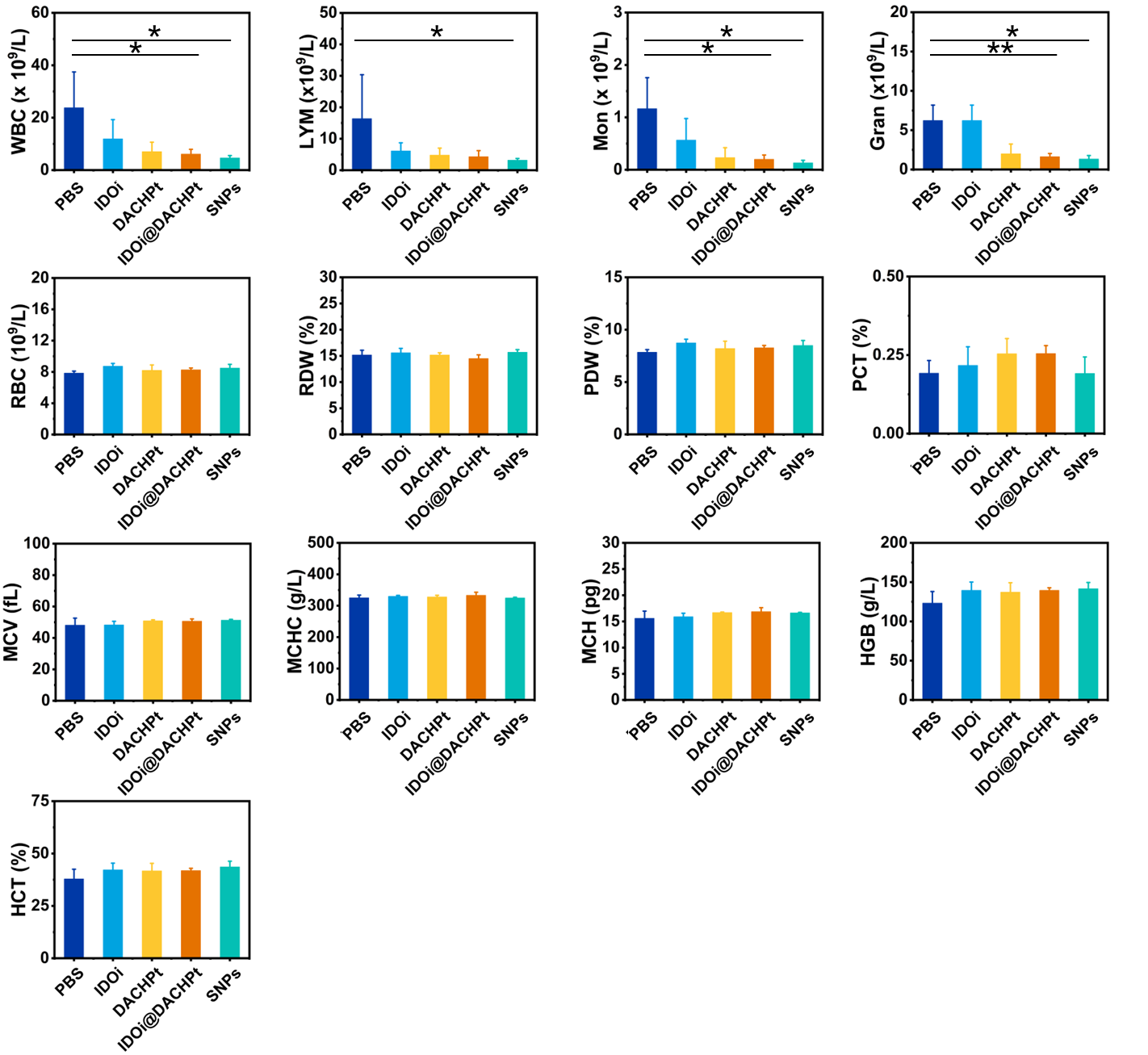


**Figure S25.** Routine blood analysis of mice after different treatments.


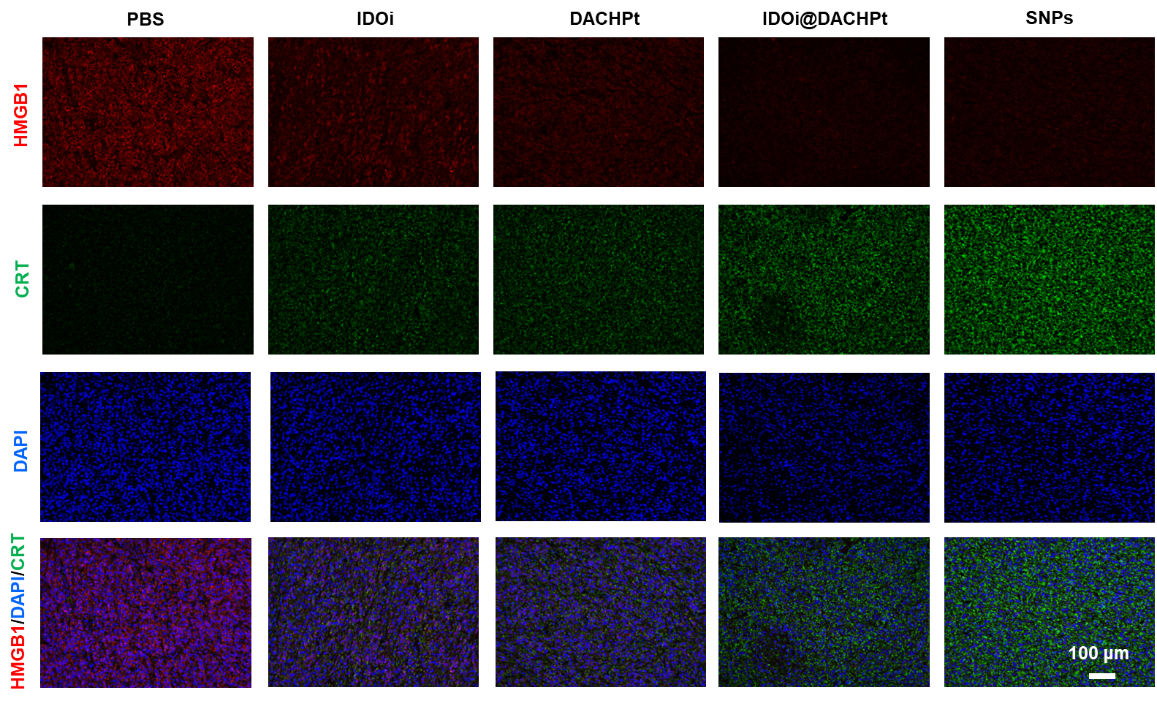


**Figure S26.** Immunofluorescence staining of HMGB1 efflux and CRT exposure in tumor tissue of mice after different treatments.


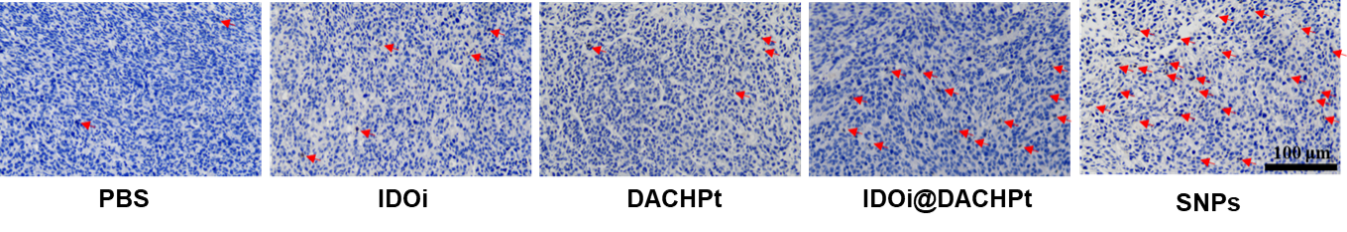


**Figure S27.** IHC staining of CD3^+^ T lymphocyte infiltration in tumor tissue of mice after different treatments.


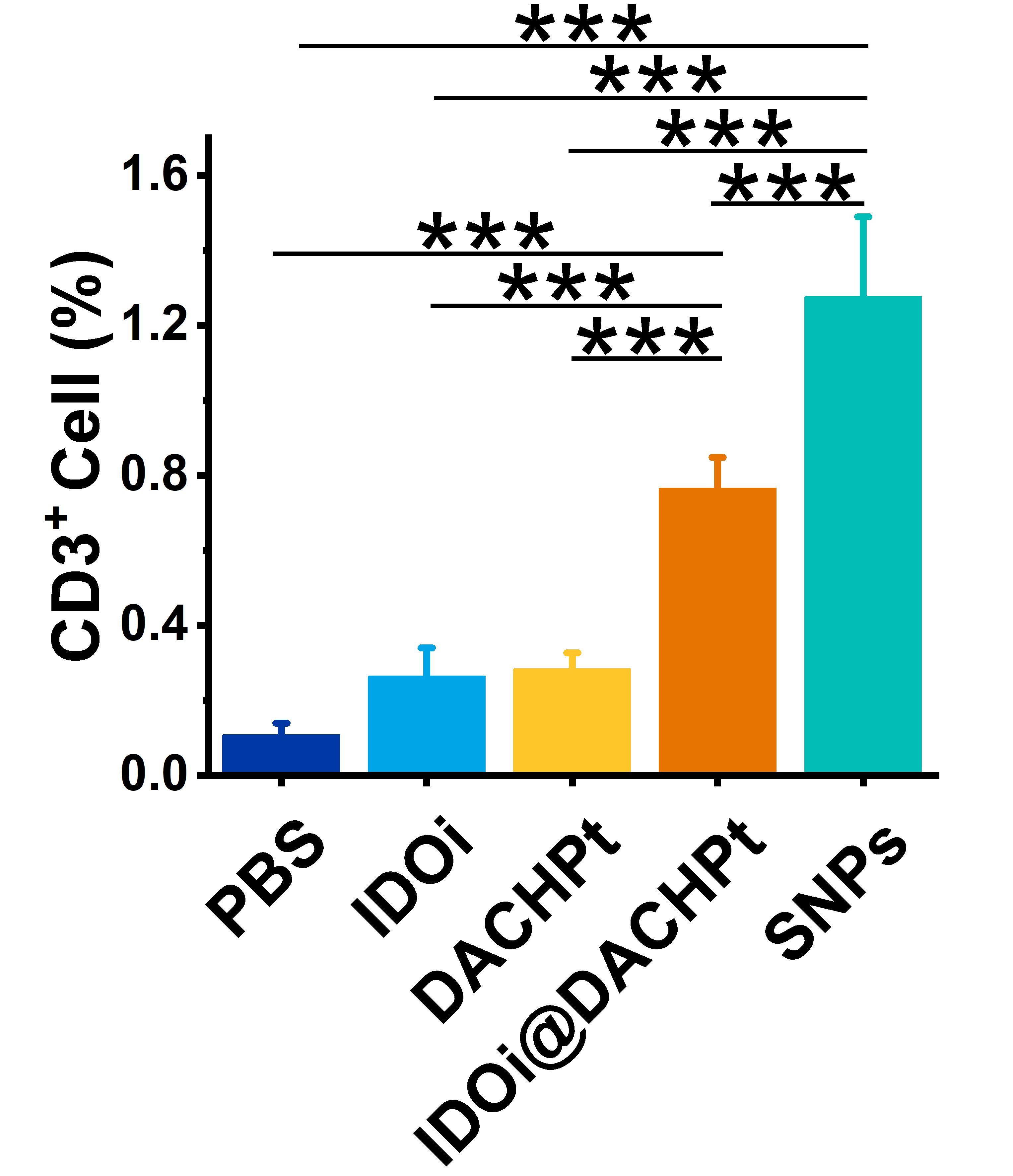


**Figure S28.** Micro-image analysis of CD3^+^ T lymphocyte infiltration in tumor tissue of mice after different treatments.


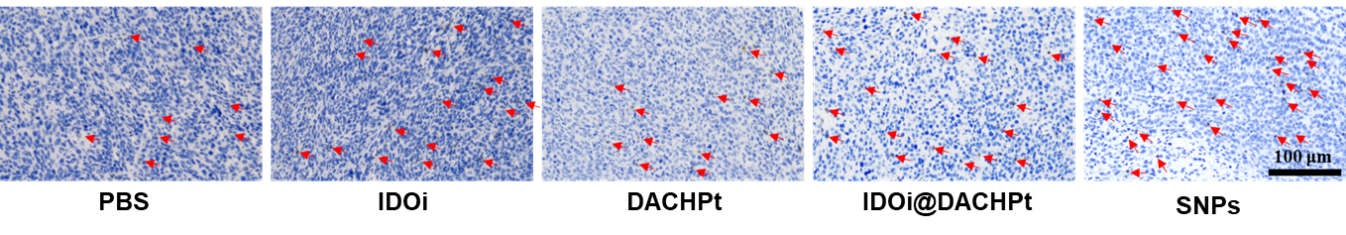


**Figure S29.** IHC staining of CD8^+^ T lymphocyte infiltration in tumor tissue of mice after different treatments.


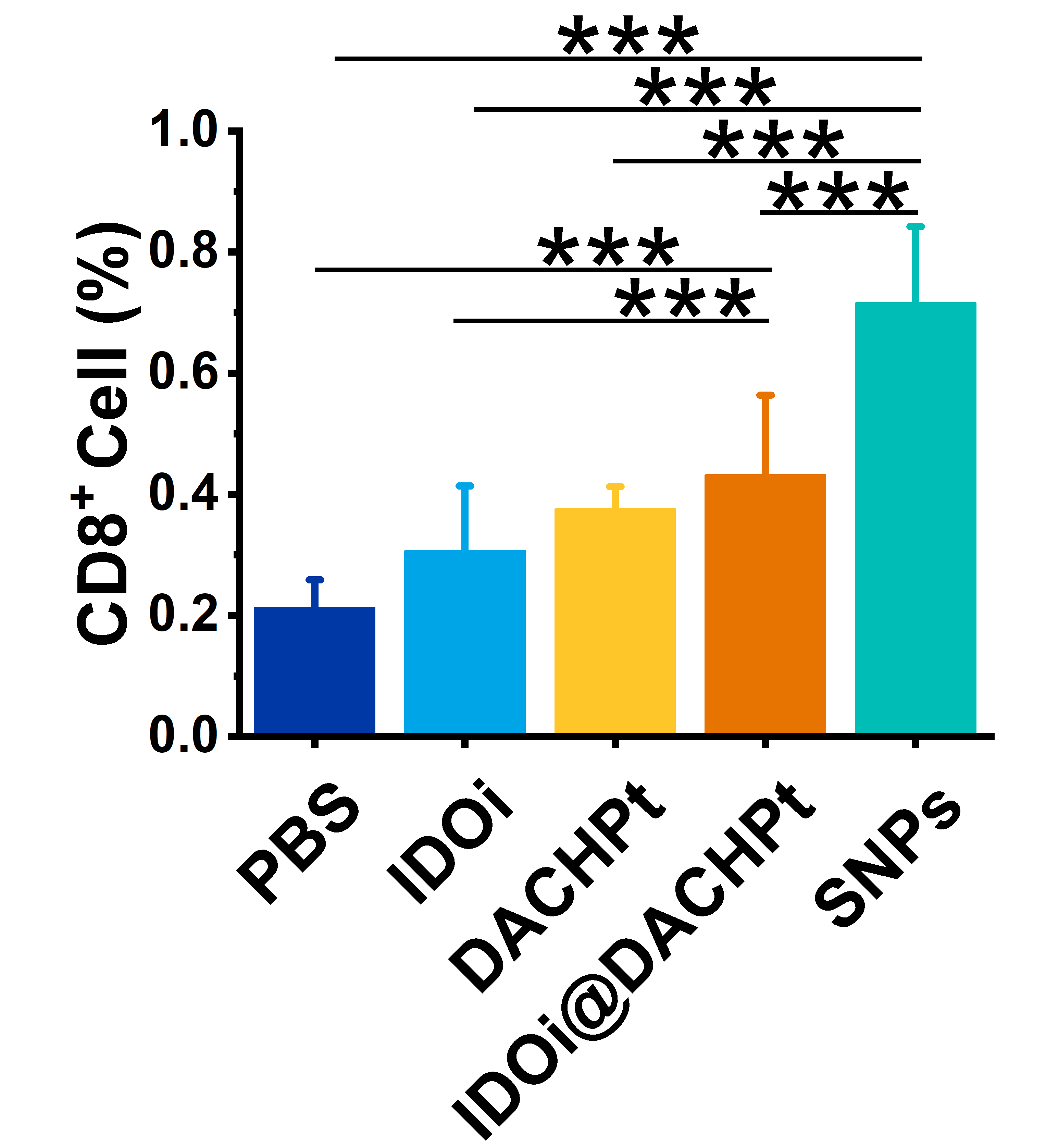


**Figure S30.** Micro-image analysis of CD8^+^ T lymphocyte infiltration in tumor tissue of mice after different treatments.


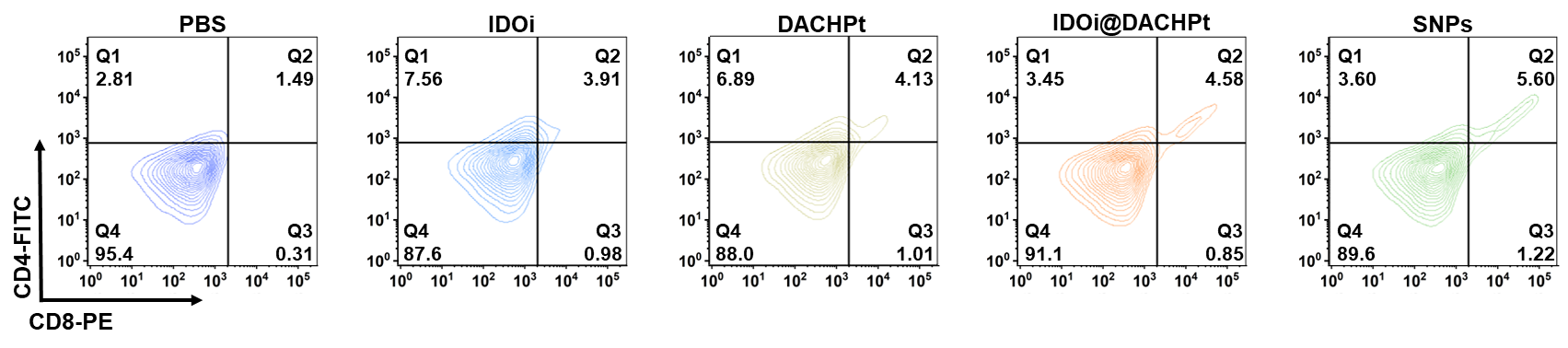


**Figure S31.** Flow cytometry analyses of CD4^+^CD8^+^ T cells in spleens of mice after different treatments.


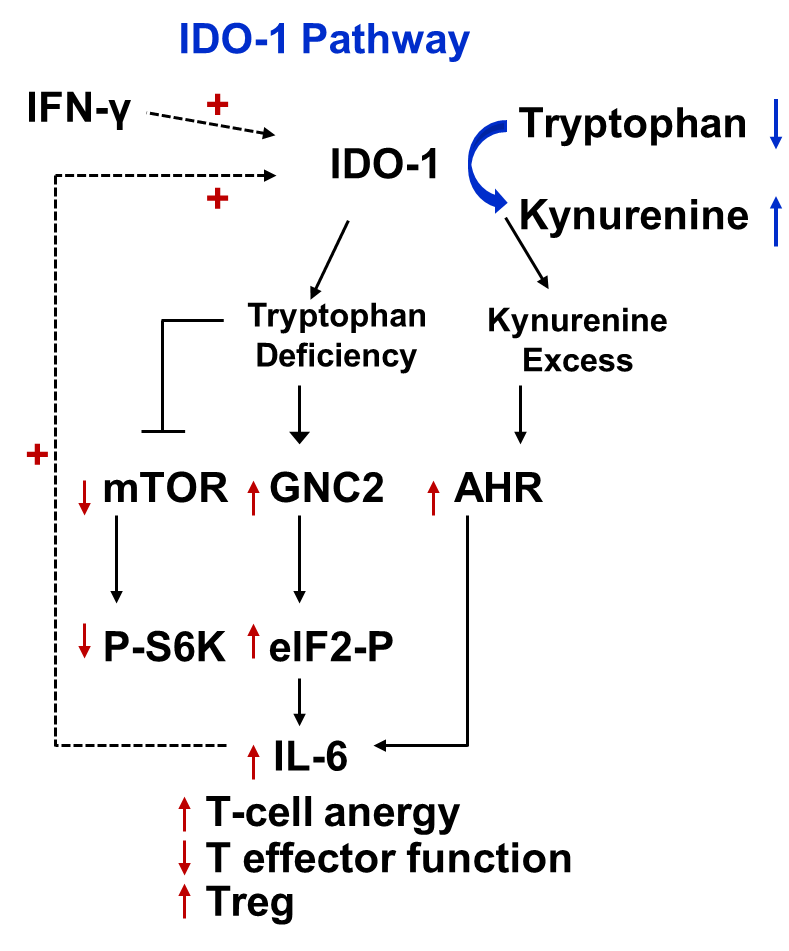


**Figure S32.** Illustration of the IDO-1 pathway.


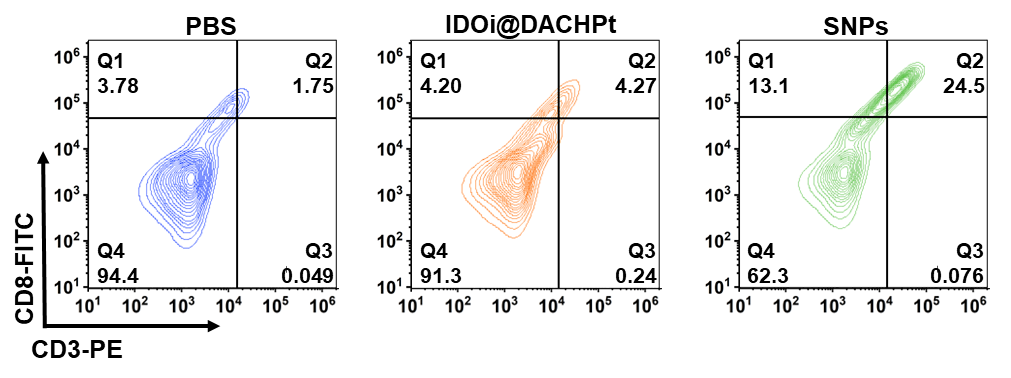


**Figure S33.** Flow cytometry analyses of CD3^+^CD8^+^ T cells in tumor tissues on the 14 day after last administration.

**Abbreviations**

IDO-1, indoleamine 2,3-dioxygenase 1; ITM, immunosuppressive tumor microenvironment; IDOi, IDO-1 inhibitor; DACHPt, 1,2-diaminocyclohexane-platinum(II); SNPs, supramolecular nanoparticles; CTLs, cytotoxicity T lymphocytes; IFN-γ, interferon-γ; Trp, tryptophan; Kyn, kynurenine; Tregs, regulatory T cells; β-CD, β-cyclodextrins; FDA, Food and Drug Administration; LiHMDS, lithium bis(trimethylsilyl)amide; ROP, ring-opening polymerization; ICD, immungentic cell death; DCs, dendritic cells; DAMPs, damage associated molecular patterns; HMGB1, high mobility group box 1; CRT, calreticulin; ATP, adenosine triphosphate; MeO-PEG-NH_2_, aminated polyethylene glycol; BLG-NCA, γ-benzyl-L-glutamate-*N*-carboxyanhydride; NOE, Nuclear Overhauser effect; ITC, isothermal titration calorimetry; *K*_a_, association constant; DLS, dynamic light scattering; TEM, transmission electron microscopy; EDX, energy-dispersive X-ray; XPS, X-ray photoelectron spectroscopy; ICP-MS, inductively coupled plasma mass spectrometry; HPLC, high performance liquid chromatography; EE, encapsulation efficiency; DLC, drug loading contents; CLSM, confocal laser scanning microscopy; M-β-CD, methyl-β-CD; PhAsO, oxophenylarsine; MTT, 3-(4′,5′-dimethylthiazol-2′-yl)-2,5-diphenyltetrazoliumbromide; IC_50_, half-maximal inhibitory concentration; OXA; oxaliplatin; EPR, enhanced permeability and retention; *t*_1/2_, blood circulation half-life; ID g^–1^, injected dose per gram; AUC, area under curve; *i.v.*, intravenous; H&E, hematoxylin and eosin; TDLNs, tumor-draining lymph nodes; ELISA, enzyme-linked immunosorbent assays; IL-6, interleukin-6, TNF-α, tumor necrosis factor-α.
